# Engineered small extracellular vesicles for targeted delivery of perlecan to stabilise the blood–spinal cord barrier after spinal cord injury

**DOI:** 10.1002/ctm2.70381

**Published:** 2025-06-19

**Authors:** Wei Peng, Wentao Zhang, Wei Cui, Wenjin Chen, Yin Zhuang, Rupeng Chu, Jinghua Tan, Jingbo Xue, Yiguo Yan, Guoyong Yin, Shujun Zhang, Yong Xie

**Affiliations:** ^1^ Department of Spine Surgery Wuxi Ninth People's Hospital Affiliated to Soochow University Wuxi China; ^2^ Wuxi Orthopedic Institute Wuxi China; ^3^ Minimally Invasive Orthopedic Institute of Soochow University Wuxi China; ^4^ Department of Cardiology The First Affiliated Hospital Hengyang Medical School University of South China Hengyang China; ^5^ Institute of Cardiovascular Disease The First Affiliated Hospital Hengyang Medical School University of South China Hengyang China; ^6^ Department of Spine Surgery The First Affiliated Hospital Hengyang Medical School University of South China Hengyang China; ^7^ Department of Orthopedics The First Affiliated Hospital of Nanjing Medical University Nanjing China; ^8^ Jiangsu Institute of Functional Reconstruction and Rehabilitation Jiangsu Provincial Clinical Research Institute Nanjing China

**Keywords:** Blood‒spinal cord barrier repair, M2 macrophage, Perlecan, RGD peptide, Small extracellular vesicles, Spinal cord injury

## Abstract

**Background:**

Destruction of the blood–spinal cord barrier (BSCB) following spinal cord injury (SCI) can result in various harmful cytokines, neutrophils, and macrophages infiltrating into the injured site, causing secondary damage. Growing evidence shows that M2 macrophages and their small extracellular vesicles (sEVs) contribute to tissue repair in various diseases.

**Methods and Results:**

In our previous proteomics‐based analysis of protein expression profiles in M2 macrophages and their sEVs (M2‐sEVs), the proteoglycan perlecan, encoded by HSPG2, was found to be upregulated in M2‐sEVs. Perlecan is a crucial component of basement membranes, playing a vital role in stabilising BSCB homeostasis and functions through its interactions with other matrix components, growth factors, and receptors. Here, we verified the high levels and remarkable therapeutic effect of M2‐sEV‐derived perlecan on the permeability of spinal cord microvascular endothelial cells exposed to oxygen glucose deprivation and reoxygenation in vitro. We also decorated the surface of M2‐sEVs with a fusion protein comprising the N‐terminus of Lamp2 and arginine glycine aspartic acid (RGD) peptides, which have an affinity for integrin αvβ3 and are primarily present on neovascular endothelium surfaces. In SCI model mice, these RGD‐M2‐sEVs accumulated at injured sites, promoting BSCB restoration. Finally, we identified M2‐sEV‐derived perlecan as a key player in regulating BSCB integrity and functional recovery post‐SCI.

**Conclusion:**

Our results indicate that RGD‐M2‐sEVs promote BSCB restoration by transporting perlecan to neovascular endothelial cells, representing a potential strategy for SCI treatment.

**Key points:**

- Perlecan, a crucial component of basement membranes that plays a vital role in stabilising BSCB homeostasis and functions, was found to be upregulated in M2‐sEVs.
- M2‐sEVs decorated with RGD peptide can effectively target the neovascular endothelium surfaces at the injured spinal cord site.
- RGD‐M2‐sEVs promote BSCB restoration by transporting perlecan to neovascular endothelial cells, representing a potential strategy for SCI treatment.

## Graphical abstract

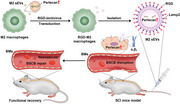

## 1 INTRODUCTION

Traumatic spinal cord injury (SCI) is a severe damage to the central nervous system (CNS) that can cause long‐term disabilities or even death, depending on the extent of injury.^1^ Based on epidemiological data from 2017, between 170 000 and 250 000 new SCI patients are diagnosed worldwide each year, indicating an SCI incidence rate of ∼23 per million in most countries.^2^ Currently, SCI is widely regarded as an incurable neurological disorder because of the lack of effective treatment strategies.

The blood–spinal cord barrier (BSCB) is a unique structure in the CNS that consists of non‐fenestrated endothelial cells, basement membranes (BMs), pericytes and astrocytic end‐foot processes.^3^, ^4^, ^5^ In contrast to peripheral circulation, intricate tight junctions (TJs), comprising zonula occludens 1 (ZO‐1), occludin and claudin 5, between adjacent endothelial cells of the BSCB are critical for limiting paracellular permeability, thereby providing a robust structural foundation for the BSCB.^6^ As a result, the BSCB functions as a relatively independent physiological entity, restricting and regulating extramedullary molecules from entering the spinal cord while maintaining a stable microenvironment for spinal cord nerve cell function.^3^, ^4^ Immediately following SCI, the BSCB and the vascular system are severely compromised^7^ by damage to the perivascular BMs. This triggers an influx of pro‐inflammatory factors and cells into the bloodstream, many of which are toxic to neurons, ultimately flooding into the spinal cord parenchyma.^8^ These changes result in secondary injuries in the parenchyma, including tissue oedema, ischaemia, hypoxia and cell death.^9^, ^10^ Furthermore, an SCI‐induced reduction in TJ proteins undermines the integrity of the BSCB.^11^ Therefore, it is imperative that we explore strategies to prevent BSCB disruption by inhibiting TJ protein degradation and restoring BSCB function, offering a potential treatment approach for SCI.

M2 macrophages are well‐established as key participants in anti‐inflammatory responses within the spinal cord epicentre during the intermediate and late stages following SCI. These macrophages are primarily responsible for promoting revascularisation, cellular proliferation and tissue growth by releasing multiple cytokines (e.g., Arg‐1, CD206, IL‐13, IL‐10 and TGF‐β), thereby playing a positive role in SCI healing.^12^, ^13^ Extracellular vesicles (EVs) contain numerous constituents derived from their parental origin, including diverse proteins, metabolites, liquids and small RNAs.^14^, ^15^ Over the past few years, researchers have studied EVs as a possible therapeutic strategy or ‘bridge’ for cell‐to‐cell communication in SCI.^16^, ^17^, ^18^ Small EVs (sEVs) derived from M2 macrophages (M2‐sEVs) were recently shown to inhibit inflammation, promote neuronal differentiation, reduce apoptosis and improve functional recovery following SCI.^19^, ^20^ However, the mechanisms underlying M2‐sEV regulation of BSCB integrity are incompletely understood, necessitating further research.

Even though the homing function of EVs may assist with the treatment of certain diseases, this targeting ability has been inefficient in animal experiments.^21^ In our previous work, the arginine‒glycine‒aspartic acid (RGD) tripeptide was quickly and efficiently conjugated to the surfaces of sEVs, improving their targeting characteristics.^17^ Further experiments confirmed that RGD‐conjugated sEVs (RGD‐sEVs) target the injured areas of the spinal cord by adhering to the integrin αvβ3 present on neovascular endothelial cells in SCI model mice.^17^ RGD‐sEVs are also reportedly effective at targeting and delivering ‘cargo’ across the blood–brain barrier (BBB) to damaged areas, effectively treating cerebral ischaemia.^22^, ^23^ Hence, to increase the concentration of cargo at post‐SCI injury sites, we developed RGD‐sEVs targeting neovascular cells.

Previously, we found that M2‐sEVs promoted post‐SCI angiogenesis and functional recovery by transporting OTU deubiquitinase with linear linkage specificity (OTULIN) protein to the endothelial cells and activating Wnt/β‐catenin signalling.^24^ In this research, we employed a proteomics‐based analysis of protein expression profiles in M2 macrophages and M2‐sEVs, revealing a marked upregulation of perlecan (*HSPG2*) that correlates well with M2‐sEV‐mediated BSCB repair.^24^ Perlecan, a component of BMs, plays a vital role in stabilising BSCB homeostasis, functioning through interactions with other matrix components, growth factors and receptors. Furthermore, perlecan has been found to contribute to functional recovery from diseases of the CNS.^25^, ^26^ Interestingly, perlecan is degraded early in injury, causing breakdown of vascular BMs and the formation of a permeable BBB with infiltration of inflammatory cells in ischaemic strokes.^27^ In SCI, perlecan degradation products recruit microglia and macrophages to the injured epicentre, where they participate in engulfing cellular debris during the early stages.^28^ Furthermore, overexpression of perlecan was shown to contribute to neural regeneration and neurological recovery following SCI.^28^ Therefore, perlecan appears to be a major contributor to maintaining and restoring BSCB functionality.

In this study, we explored the role of M2‐sEVs in restoring the BSCB following SCI. First, we verified our previous findings of high levels of perlecan in M2‐sEVs in a proteomics‐based analysis showing perlecan upregulation in M2‐sEVs over M2 macrophages. Next, we fused the RGD peptide to Lamp2 on the surface of M2‐sEVs. In vivo experiments demonstrated that RGD‐decorated M2‐sEVs targeted the newly formed vascular endothelial cells at the injured sites in SCI mice, showing restorative effects on BSCB. Finally, we identified a key role for perlecan in the M2‐sEV‐mediated regulation of BSCB integrity and neurological recovery following SCI.

## 2 MATERIAL AND METHODS

### 2.1 Cell culture

As previously described, bone marrow‐derived macrophages (BMDMs) were isolated.^24^ After stimulating the macrophages with 30 ng/mL macrophage colony‐stimulating factor (M‐CSF) for 5–7 days, the culture medium was switched to an M2 macrophage medium (IMDM; Gibco) supplemented with 10 ng/mL IL‐4 (Peprotech), 10% foetal bovine serum (FBS; Gibco) and 1% antibiotic‒antimycotic (Gibco) for 3 days to enhance macrophage proliferation. Control group was unstimulated macrophages (M0).

As described by Ruck et al. in 2014, primary spinal cord microvascular endothelial cells (SCMECs) were isolated and cultured.^29^ Briefly, the spinal cord from a female C57BL/6 mouse aged 8–12 weeks was extracted, minced and digested with collagenase type II. Myelin was removed from the resulting cell pellet using bovine serum albumin (BSA) in Dulbecco's modified Eagle's medium (DMEM; 20%, w/v), followed by a second digestion with 1% collagenase/dispase. Finally, microvascular segments were washed twice and seeded in low‐glucose DMEM supplemented with 20% FBS,0.5% basic fibroblast growth factor,1% heparin and 1% puromycin on six‐well plates coated with collagen I (Sigma). Cell culture medium was only supplemented with puromycin for the first 2 days of culture.

### 2.2 Construction and transduction of lentivirus vectors expressing RGD‐Lamp2 and perlecan‐targeting short hairpin RNA

A lentivirus vector expressing gcGFP‐RGD‐tag‐Lamp2 was constructed and packaged (sense: TGTCGTGGTGATAAAGGTCCAGATTGT; antisense: ACAGCACC ACTATTTCCAGGTCTAACA) by Genechem Co., Ltd. Following the manufacturer's instructions, we transduced the gcGFP‐RGD‐tag‐Lamp2 expression vector into M0 and M2 macrophages. Puromycin was then employed to remove non‐transduced macrophages and obtain pure populations of RGD‐M0 and RGD‐M2 macrophages. We also conducted a control experiment in which scrambled RGD (Scr) peptides were conjugated to M2 macrophages (Scr‐M2).

For perlecan knockdown, Cyagen Biosciences provided three lentivirus‐expressing short hairpin RNA (shRNA) constructs (ShPerlecan #1, ShPerlecan #2 and ShPerlecan #3) and scrambled control shRNA (Con shRNA), along with virus packaging services. Cells were transduced according to the manufacturer's instructions.

### 2.3 sEV isolation and identification

The supernatant was collected from M2 macrophages, following two washes with phosphate‐buffered saline (PBS) and 48 h of culture in a medium containing exosome‐free FBS (VivaCell Biosciences). Based on Théry et al.’s 2006 study, the macrophage‐derived sEVs were isolated using differential centrifugation from conditioned medium.^30^ Briefly, we centrifuged the finished medium containing exosome‐free FBS for 10 min at 4°C at a speed of 300 ×*g* to remove any cell debris. The cleared supernatant was then centrifuged again at 4°C and 2000 ×*g* for 10 min to discard dead cells. As a next step, the supernatant was transferred to a specialised tube for use in an ultracentrifuge and spun at 4°C for 30 min at 10 000 ×*g*. The supernatant was passed through a 0.22 µm filter (Millipore) and then ultracentrifuged at 4°C and 140 000 ×*g* for 3 h, resulting in pellets containing sEVs.

Transmission electron microscopy (TEM; Hitachi) evaluated the sEV morphology. Nanoparticle tracking analysis measures the size and number of sEVs. Each group of sEVs contained five independent samples, and four clear‐field microscopy views from each sample were randomly selected for analysis. Immunoblotting for the cell marker calnexin and the sEV markers CD81, CD63 and TSG101 were performed using antibodies from Abcam. The concentration of sEVs was measured using the bicinchoninic acid (BCA) method. For in vitro assays, complete medium served as the Vehicle control, while in vivo studies were conducted using PBS as the control.

### 2.4 Animals and surgical procedures

The Wuxi Ninth Affiliated Hospital of Soochow University's Ethics Committee for Scientific Research approved all animal experiments (KS2024057). The mice were maintained in a climate‐controlled setting with a 22°C–24°C temperature, 60%–80% humidity, 12‐h cycle of daylight and darkness, and sufficient water and food. For SCI modelling, a 50:5:1 dose of ketamine, xylazine and acepromazine (50%:50%) was administered intraperitoneally to female C57BL/6 mice to achieve a deep anaesthesia. After that, the 10th thoracic vertebral spinal cord was exposed and injured by a modified Allen's weight‐drop impactor, which involved a weight of 10 g dropping from a vertical height of 20 cm. The success of SCI modelling was primarily assessed through Basso Mouse Scale (BMS) scores and subscores, and histological evaluation using haematoxylin and eosin staining. In sham mice, only the laminae were removed, with no damage to the spinal cord. A bladder massage was conducted to facilitate urination, followed by penicillin sodium administration (North China Pharmaceutical) for 3 days following the surgery. Immediately following SCI and every 24 h thereafter, RGD‐M0‐sEVs (RGD‐blank sEVs), Scr‐M2‐sEVs, RGD‐M2‐sEVs (100 µg per 100 µL) or Vehicle (100 µL) were injected into the tail vein.

### 2.5 Tracking macrophage‐derived sEVs in vivo

To track macrophage‐derived sEVs in vivo, the fluorescent lipophilic tracers Dil (Sigma) or DiR (Yeasen Biotechnology) were used. The DiR‐labelled sEVs were administered through the tail vein instantly following SCI, and the mice were euthanised at 7 days post‐SCI to collect brain, spinal cord, lung, heart, liver, spleen, kidney, stomach and intestinal tissues. The biodistribution of labelled sEVs in these organs was analysed using a near‐infrared fluorescence imaging facility (NIRF; Perkin Elmer). The uptake of Dil‐labelled sEVs administered via intravenous injection through the tail vein instantly following SCI and then every 24 h, lasting 3 days (100 µg per 100 µL), was measured using immunofluorescence. On day 7 post‐SCI, spinal cord tissues were collected for frozen sections.

### 2.6 Oxygen glucose deprivation and reoxygenation

As described by Liu et al. in 2012, SCMECs were exposed to oxygen glucose deprivation (OGD) to replicate the hypoxic ischaemic state in vivo.^31^ Briefly, SCMECs were grown in glucose‐free DMEM (Gibco) on Petri dishes in a humidified anaerobic chamber (5% CO_2_ and 95% N_2_ with 0.2% O_2_) for 6 h, then transferred to standard culture conditions under normoxia for 24 h of reoxygenation.

### 2.7 Evans blue dye assays

To investigate BSCB permeability, we performed an extravasation assay with Evans blue (EB) dye (Sigma‒Aldrich), as described by Ge et al. in 2021.^32^ Briefly, 1 mL of EB dye was administered to the tail vein at various intervals following SCI. EB leakage was assessed from digital photographs of spinal cord taken on day 7 post‐SCI. For the final step of the analysis, the spinal cord tissues were collected to prepare frozen sections for evaluation under fluorescence microscopy (Zeiss Apotome 3). ImageJ (NIH) was used to quantify the results.

### 2.8 Trans‐endothelial permeability assay

Trans‐endothelial permeability was measured with FITC‐dextran. Briefly, upon reaching confluence, SCMECs were seeded into a 24‐well Transwell chamber with a 0.4 µm filter (Corning) at a density of 2 × 10^5^ cells/mL. Following the OGD treatment, a 1 mg/mL solution of 40‐kDa FITC‐dextran (Sigma) was added to the upper chamber, while the lower chamber was filled with PBS. After 1 h of light shielding in the lower chamber, an optical microplate reader (Thermo Fisher Scientific) was employed to quantify 490 and 520 nm fluorescence intensities. PBS was used as the negative control, and the initial FITC‐dextran solution in the upper chamber as the positive control. FITC‐dextran transport (%) = (lower chamber medium fluorescence intensity ‒ negative control)/positive control.

### 2.9 Evaluation of locomotor function

Basso et al. described a system of BMS scores used to assess mouse motor function in 2006.^33^ It consists of nine levels (complete paralysis to normal locomotion) and an 11‐point subscore including plantar pedaling frequency, bilateral hindlimb coordination, paw position, trunk stability and tail position. Two blinded investigators observed each mouse for 5 min, during which time the average BMS scores and subscores were recorded.

### 2.10 Neuroelectrophysiology

The motor evoked potentials (MEPs) were recorded using the Schlag et al. 2021 methodology.^34^ In brief, an electrode was attached to the area of the skull adjacent to the motor cortex, a recording electrode was implanted into the anterior tibialis muscle of the opposite hindlimb, and a reference electrode was positioned between the recording and stimulation electrodes within the subcutaneous tissue. The amplitude and latency of the mean MEP values were collected pre‐surgery (baseline) and at 56 days post‐surgery (post‐surgical). The tested MEPs (mV) represent the relative level of recovered motor function in individual mice.

### 2.11 Quantitative real‐time PCR analysis

TRIzol reagent (Invitrogen) was used to extract total RNA. Single‐stranded cDNA was reverse transcribed from 1 g of total RNA for each sample using the GoScript Reverse Transcription System (Promega Corporation). The quantitative real‐time PCR (qRT‐PCR) was carried out with GoScript qPCR Master Mix (Promega), utilising an FTC‐3000 real‐time PCR system (Funglyn Biotech Inc.) to process and analyse all reactions. An analysis of relative gene expression has been performed utilising the 2^–ΔΔCt^ method, with *GAPDH* expression normalised to that of the target gene. The qRT‐PCR primer sequences are shown in Table 1.

**TABLE 1:** Gene‐specific primer sequences used for quantitative real‐time PCR (qRT‐PCR).

Claudin‐5	Forward primer	GCAAGGTGTATGAATCTGTGCT
	Reverse primer	GTCAAGGTAACAAAGAGTGCCA
Occludin	Forward primer	TGGGCAGTCGGGTTGACT
	Reverse primer	GGGCATCATGGTGTTCATTG
ZO‐1	Forward primer	GCCGCTAAGAGCACAGCAA
	Reverse primer	TCCCCACTCTGAAAATGAGGA
Perlecan	Forward primer	TGGAGCCCGAATACAGGAAGA
	Reverse primer	AGATCCGTCCGCATTCCCT

### 2.12 Western blotting

Total protein extracts from each sample were obtained with RIPA buffer containing protease and phosphatase inhibitors (Sigma). Supernatant was harvested following a 10‐min centrifugation, and protein concentration was measured with a BCA protein assay kit (Beyotime). Proteins were electrophoresed using sodium dodecyl sulphate‒polyacrylamide gel (20 g per sample) and subsequently transferred to PVDF membranes (Millipore). Following blocking with 5% milk in Tris‐buffered saline containing Tween 20 (TBST) for 60 min at room temperature (20°C‒25°C), the membranes were incubated with primary antibodies overnight at 4°C. Table S1 provides details about the primary antibodies and dilutions that were used here. After a TBST rinse, the blots were incubated with goat anti‐rabbit IgG conjugated with peroxidase (1:5000 dilution; CST). To verify equal protein loading, a rabbit anti‐actin antibody (1:5000, CST) was employed as a control. After enhancement with Thermo Fisher Scientific chemiluminescence reagents, the data were analysed with a ChemiDoc luminescent image analyser (Bio‐Rad).

### 2.13 Immunofluorescence

A cryostat (Thermo Fisher Scientific) was employed to cut sections (20‐mm thickness) of mouse spinal cord. Three PBS washes were followed by permeabilisation with.5% Triton X‐100 for 30 min, blocking with 5% BSA for 1 h, and immunolabeling overnight with primary antibodies (see Table S1 for detailed information on the primary antibodies). The secondary antibody was incubated at room temperature for 1.5 h, followed by washing the sections in PBS. A final step in the mounting process utilised Fluoroshield with DAPI (GeneTex Inc.). Cell samples (five per group) were fixed with 4% paraformaldehyde prior to the immunolabeling procedure described above. Each sample was imaged using fluorescence microscopy (Zeiss Apotome 2), capturing five field views at 200× magnification, with each field measuring of 600 × 500 µm. ImageJ was used to quantify the images.

For in vitro data, Zeiss Apotome 2 was used to calculate the mean fluorescence intensity quantitatively. For in vivo data, fluorescence intensity was measured using ImageJ software in five randomly chosen injured areas. To quantify the fluorescence of TJ proteins (ZO‐1, Occludin and Claudin‐5), we determined the mean fluorescence intensity after segmenting the cell membrane using the MorphoLibJ plugin.

### 2.14 Statistical analysis

Data were statistically evaluated using SPSS 22.0 (IBM Corp.) and are displayed as means and standard errors. An unpaired *t*‐test was conducted to compare the two groups. To compare three or more groups, or to analyse groups over time, one‐way or two‐way analysis of variance with Tukey's post hoc test was employed. A *p*‐value <.05 was considered statistically significant.

## 3 RESULTS

### 3.1 M2 macrophages and M2‐sEVs exhibit high expression of perlecan, with M2‐sEVs decreasing the permeability of OGD‐treated SCMECs in vitro

**FIGURE 1:**
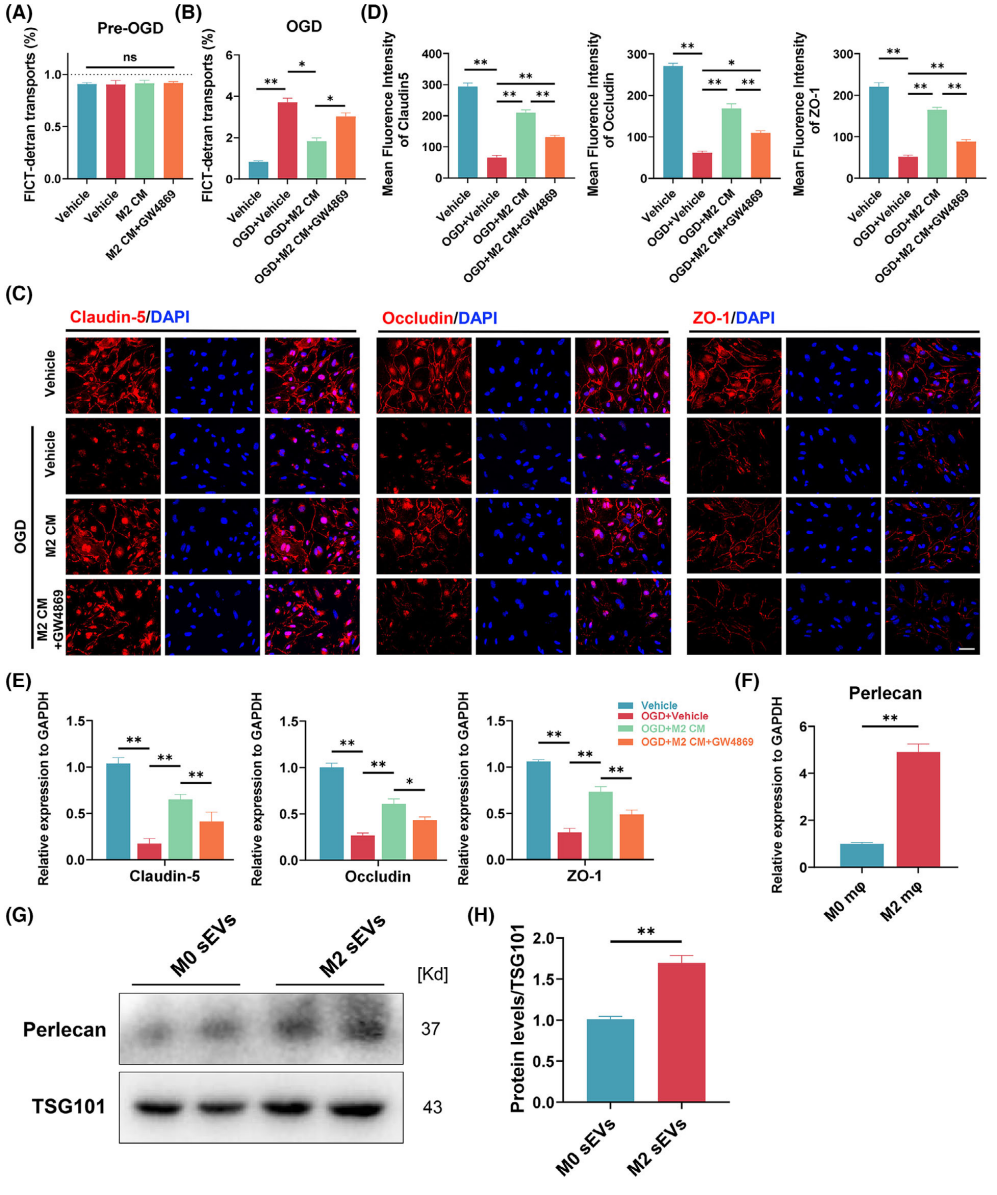
M2 macrophage and its small extracellular vesicles (sEVs) were highly expressed perlecan, and M2 sEVs decreased the permeability of spinal cord microvascular endothelial cells (SCMECs) treated with oxygen glucose deprivation (OGD) in vitro. (A and B) FITC‐dextran transports analysed the permeability of SCMECs pre‐ and post‐OGD after treatment with Vehicle, M2 CM or M2 CM + GW4869. *n* = 5 per group. (C) Immunofluorescence images of tight junction‐related proteins (Claudin‐5, Occludin and ZO‐1) in SCMECs treated with Vehicle, M2 CM or M2 CM + GW4869 after exposure to OGD. Scale bar, 20 µm. (D) Quantification evaluation of the fluorescence intensity of Claudin‐5, Occludin and ZO‐1 in (C). *n* = 5 per group. (E) mRNA expressions of Claudin‐5, Occludin and ZO‐1 in SCMECs after treatment with Vehicle, M2 CM, or M2 CM + GW4869 pre‐ and post‐OGD. *n* = 5 per group. (F) Quantitative real‐time PCR (qRT‐PCR) verified the mRNA expressions of perlecan in M0 and M2 macrophages. *n* = 5 per group. (G) Western blotting analysis of perlecan protein levels in M0 and M2 sEVs. *n* = 3 per group. (H) Quantification perlecan expression levels of (G). *n* = 3 per group. ns, no significance, ^*^ *p* < .05, ^**^ *p* < .01. Statistical tests were performed using one‐way analysis of variance (ANOVA) with Tukey's post hoc test (A‒E) and unpaired *t*‐test (F‒H).

In our previous study, we performed a proteomics‐based analysis of protein expression profiles in M2 macrophages and M2‐sEVs.^24^ We observed a marked upregulation of perlecan (*HSPG2*) in M2‐sEVs, positively correlating with BSCB repair. To explore whether M2‐sEVs contribute to improved SCMEC permeability, we first examined the SCMEC‐mediated transfer of FITC‐dextran pre‐ and post‐exposure to OGD. The results showed that, before OGD, the FITC‐dextran transport rate was below 1% in all SCMEC groups, with no significant between‐group differences (Figure 1A). By contrast, SCMEC‐mediated FITC‐dextran transport was significantly elevated after OGD, with a strong diminishment in this trend under M2 culture medium (M2 CM). The promotional effect of M2 CM on SCMEC permeability was blocked when the OGD + M2 CM group was treated with GW4869, which inhibits the cellular release of sEVs (Figure 1B). Consistently, immunofluorescence staining showed that the fluorescence intensity of TJ‐associated proteins (Claudin‐5, Occludin and ZO‐1) in SCMECs was decreased under OGD but was increased under M2 CM treatment, with GW4869 appearing to inhibit this phenomenon (Figure 1C,D). Furthermore, qRT‐PCR analysis confirmed that M2 CM increased the levels of mRNA for Claudin‐5, Occludin and ZO‐1 after treatment with OGD, which was inhibited by GW4869 (Figure 1E). These findings indicated that M2‐sEVs may contribute to improving SCMEC permeability.

To confirm the enrichment of perlecan in M2‐sEVs, we performed qRT‐PCR analysis of *HSPG2* mRNA levels in M2 macrophages. As shown in Figure 1F, *HSPG2* mRNA expression was remarkably higher in M2 macrophages than in M0 macrophages. Next, Western blotting analysis indicated that, compared with un‐stimulated macrophage sEVs (M0‐sEVs), M2‐sEVs expressed a high level of HSPG2, suggesting that the M2‐sEVs were enriched in perlecan (Figure 1G,H). These results demonstrated that M2 macrophages and M2‐sEVs exhibit high levels of HPSG2, indicating that M2‐sEVs may reduce SCMEC permeability under exposure to OGD in vitro.

### 3.2 Preparation of RGD‐M2‐sEVs, which target neovascular endothelial cells post‐SCI

**FIGURE 2:**
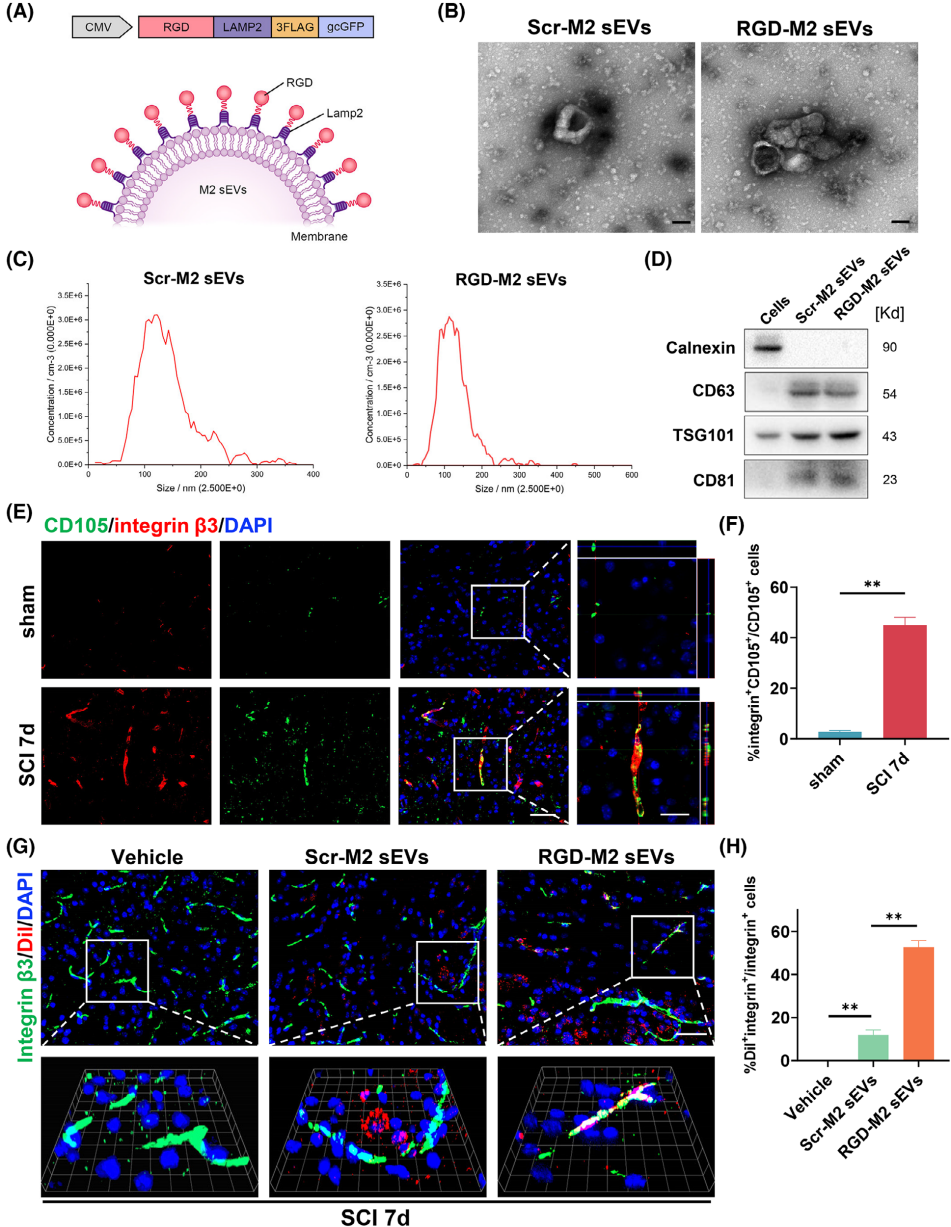
Preparation and characterisation of RGD‐M2 small extracellular vesicles (sEVs) and their ability to target neovascular endothelial cells post‐spinal cord injury (SCI). (A) Schematic diagram illustrates the structure of targeted sEVs derived from RGD‐M2 macrophages. (B) Image representations of Scr‐M2 and RGD‐M2 transmission electron microscopy (TEM) images. Scale bar, 100 nm. (C) Nanoparticle tracking analysis (NTA) of Scr‐M2 and RGD‐M2 sEV diameter and particle number. (D) Western blotting analysis of sEVs specific surface markers (CD63, TSG101 and CD81) and cellular marker (Calnexin). (E) Immunofluorescence co‐staining of CD 105 (neovascular endothelial cell, green) and integrin β3^+^ cells (red) in the injured epicentre on sham and day 7 after SCI. Scale bar, 50 and 20 µm (enlarged view). (F) Analysing the proportion of integrin β3^+^CD105^+^ cells in CD105^+^ cells in (E). (G) Immunofluorescence images of integrin β_3_^+^ cells (green) and Dil‐labelled sEVs (red) at the injured site at sham and 7‐day after SCI. Scale bar, 50 and 20 µm (enlarged view). (H) Analysing the proportion of Dil^+^integrin β_3_^+^ cells in integrin β_3_^+^ cells in (G). *n* = 5 per group. Bars indicate mean ± SEM. ^**^ *p* < .01 compared with Sham surgery or Vehicle group. Statistical tests were performed using unpaired *t*‐test (E and F) and one‐way analysis of variance (ANOVA) with Tukey's post hoc test (G and H).

EVs administered via systemic injection cannot effectively target and carry cargo to the epicentre region. Therefore, we engineered a targeted delivery system by linking the RGD peptide to the surface of M2‐sEVs. First, BMDMs were matured and polarised into M2 macrophages via stimulation with M‐CSF and IL‐10. Next, a lentivirus encoding pgcGFP‐RGD‐Lamp2 was transduced into M2 macrophages to fuse the RGD peptide specific to neovascular endothelial cells with the Lamp2 N‐terminus on the sEVs outer membrane, as shown in Figure 2A. After purifying the transduced M2 macrophages via puromycin selection, they were transduced with RGD‐Lamp2, and the sEVs were purified by ultracentrifugation separation and characterised. Figure 2B,C illustrates that both RGD‐M2‐sEVs and control Scr‐M2‐sEVs were cup‐ or sphere‐shaped, with identical nanoparticle size distributions, similar to our previous.^35^ In Western blotting analyses, specific sEV biomarkers, such as TSG101, CD63 and CD81, and the cellular marker calnexin, were detected (Figure 2D).

Before assessing the targetability of RGD‐M2‐sEVs, it was critically important to determine whether there was high expression of integrin β3 in the neovascular endothelium of the injured region of the spinal cord. Immunofluorescence analysis revealed only slight CD105 fluorescence in the spinal cord prior to injury (Figure S1A,B). By contrast, at day 7 post‐SCI, CD105 fluorescence intensity was vigorous and primarily co‐stained with CD31 in the injured epicentre, indicating that CD105 effectively labelled neovascular cells in the injured area. Furthermore, the fluorescence of integrin β3, an exclusive partner of integrin αvβ3, predominantly overlapped with CD105^+^ cells at the injured site (Figure 2E,F). These results suggested that integrin αvβ3 may be primarily and strongly expressed in the neovascular endothelium in the injured region following SCI, in agreement with previous research.^36^

To confirm the ability of RGD‐M2‐sEVs to target neovascular endothelium in the lesion, we intravenously injected a 100‐µL volume (1 µg/µL) of Dil‐labelled M2‐sEVs conjugated with RGD or Scr peptides, or Vehicle control, into SCI model mice. Immunofluorescence images showed the accumulation of some Dil‐labelled RGD‐M2‐sEVs within integrin β_3_^+^ cells, with less overlap between Dil‐labelled Scr‐M2‐sEVs and integrin β_3_ (Figure 2G,H). Additionally, to explore whether the RGD peptide improved the tropism of M2‐sEVs to the injured area, we injected DiR‐labelled RGD‐M2‐sEVs or Scr‐M2‐sEVs into the tail vein after SCI. NIRF imaging was then utilised to analyse fluorescence in the brain, heart, lungs, spinal cord, liver, spleen, kidneys, stomach and intestines of these mice for 4 h following injection (Figure S2A). We found that DiR fluorescence of Scr‐M2‐sEVs accumulated primarily in the liver, spleen, stomach and lungs, but was not detected within injured spinal cords. In contrast, the DiR fluorescence RGD‐M2‐sEVs was remarkably elevated in the spinal cord (Figure S2B,C). Analysis of the DiR level in other organs (excluding the spinal cord) revealed an increase in the stomach of the RGD‐M2‐sEV group compared with that of the Scr‐M2‐sEV group (Figure S2D). Importantly, although the RGD‐M2‐sEV group also showed slightly higher DiR fluorescence in the liver and spleen than the Scr‐M2‐sEV group, no significant differences in fluorescence expression were detected in the other seven organs, highlighting the targeting ability of the RGD‐M2‐sEVs. These results indicated that M2‐sEVs decorated with RGD peptide showed an improved ability to target the epicentre of SCI lesions.

### 3.3 RGD‐M2‐sEVs improve the permeability of SCMECs exposed to OGD in vitro

**FIGURE 3:**
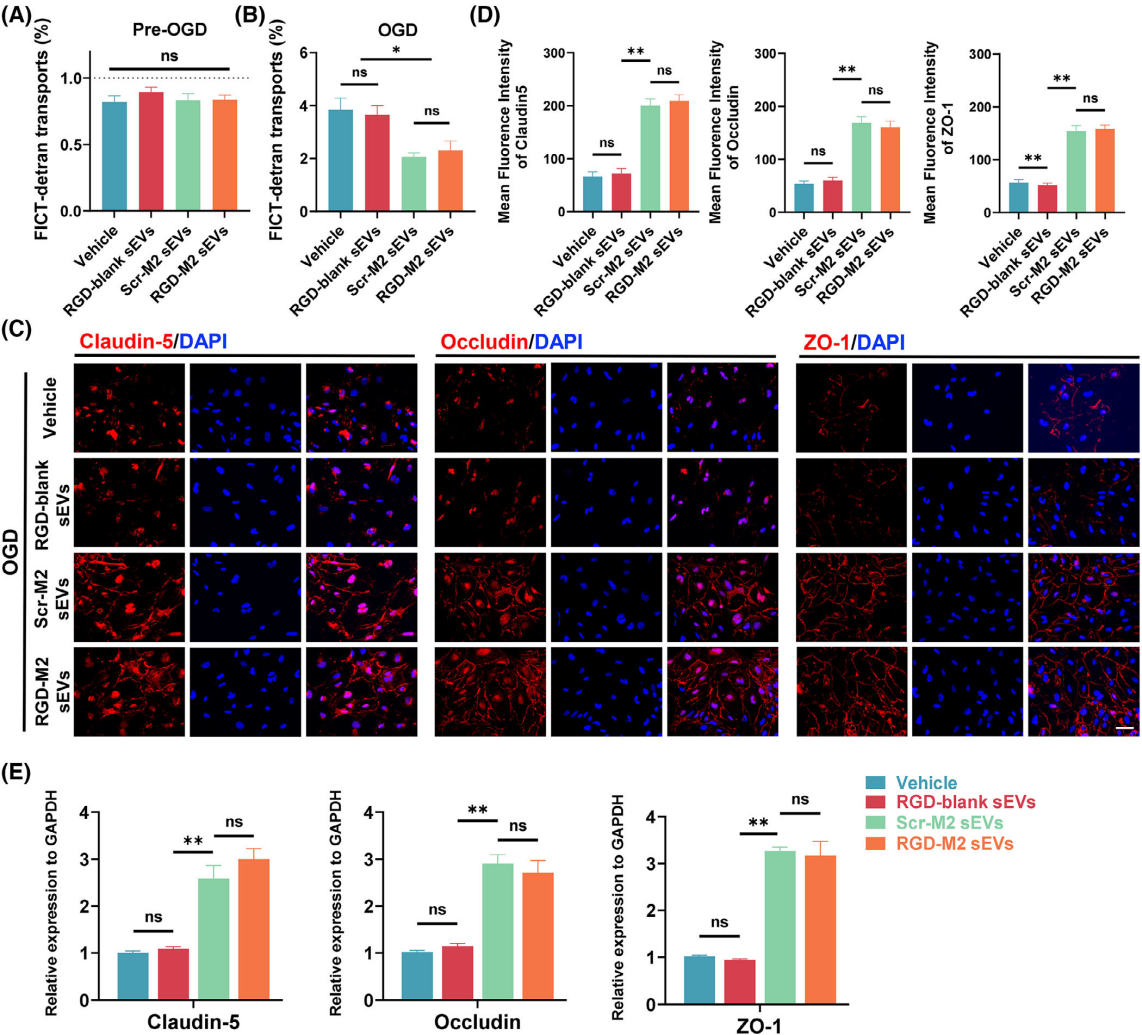
RGD‐M2 small extracellular vesicles (sEVs) improved the spinal cord microvascular endothelial cells (SCMECs) permeability after exposure to oxygen glucose deprivation (OGD) in vitro. (A and B) FITC‐dextran transport assay examines SCMEC's permeability in the presence of Vehicle, RGD‐blank sEVs, Scr‐M2 sEVs or RGD‐M2 sEVs pre‐ and post‐OGD. *n* = 5 per group. (C) Immunofluorescence images of tight junction‐related proteins (Claudin‐5, Occludin and ZO‐1) when OGD‐exposed SCMECs were treated with Vehicle, RGD‐blank sEVs, Scr‐M2 sEVs or RGD‐M2 sEVs. Scale bar, 20 µm. (D) Analysing the fluorescence intensity of Claudin‐5, Occludin and ZO‐1 in (C). *n* = 5 per group. (E) qPCR verification of the mRNA levels of Claudin‐5, Occludin and ZO‐1 in SCMECs treated with Vehicle, RGD‐blank sEVs, Scr‐M2 sEVs or RGD‐M2 sEVs post‐OGD. *n* = 5 per group. ns, no significance, ^*^ *p* < .05, ^**^ *p* < .01. Statistical tests were performed using one‐way analysis of variance (ANOVA) with Tukey's post hoc test (A‒E).

To investigate the influence of RGD‐M2‐sEVs on permeability in vitro, we first evaluated SCMECs' ability to transport FITC‐dextran in an OGD model. The results demonstrated that the rate of FITC‐dextran transport in all SCMEC groups was below 1% before exposure to OGD, with no significant between‐group differences. After exposure to OGD, FITC‐dextran transport remarkably increased, but was thwarted by treatment with Scr‐M2‐sEVs or RGD‐M2‐sEVs (Figure 3A,B). Immunofluorescence analysis showed minimal fluorescence of TJ proteins in OGD‐exposed Vehicle and RGD‐blank groups. By contrast, the Scr‐M2‐sEV and RGD‐M2‐sEV treatment groups showed dramatic increases in the fluorescence intensity of these TJ proteins under OGD exposure (Figure 3C,D). Consistently, qRT‐PCR analysis showed that, while there were minor differences in the mRNA levels of these TJ proteins in the Vehicle and RGD‐blank groups, all three were upregulated following Scr‐M2‐sEV or RGD‐M2‐sEV treatment in the OGD model (Figure 3E). These findings demonstrated that both Scr‐M2‐sEVs and RGD‐M2‐sEVs enhanced SCMEC permeability in the OGD model in vitro, with RGD‐M2‐sEVs providing superior efficacy.

### 3.4 M2‐sEVs and RGD‐M2‐sEVs restore post‐SCI BSCB integrity

**FIGURE 4:**
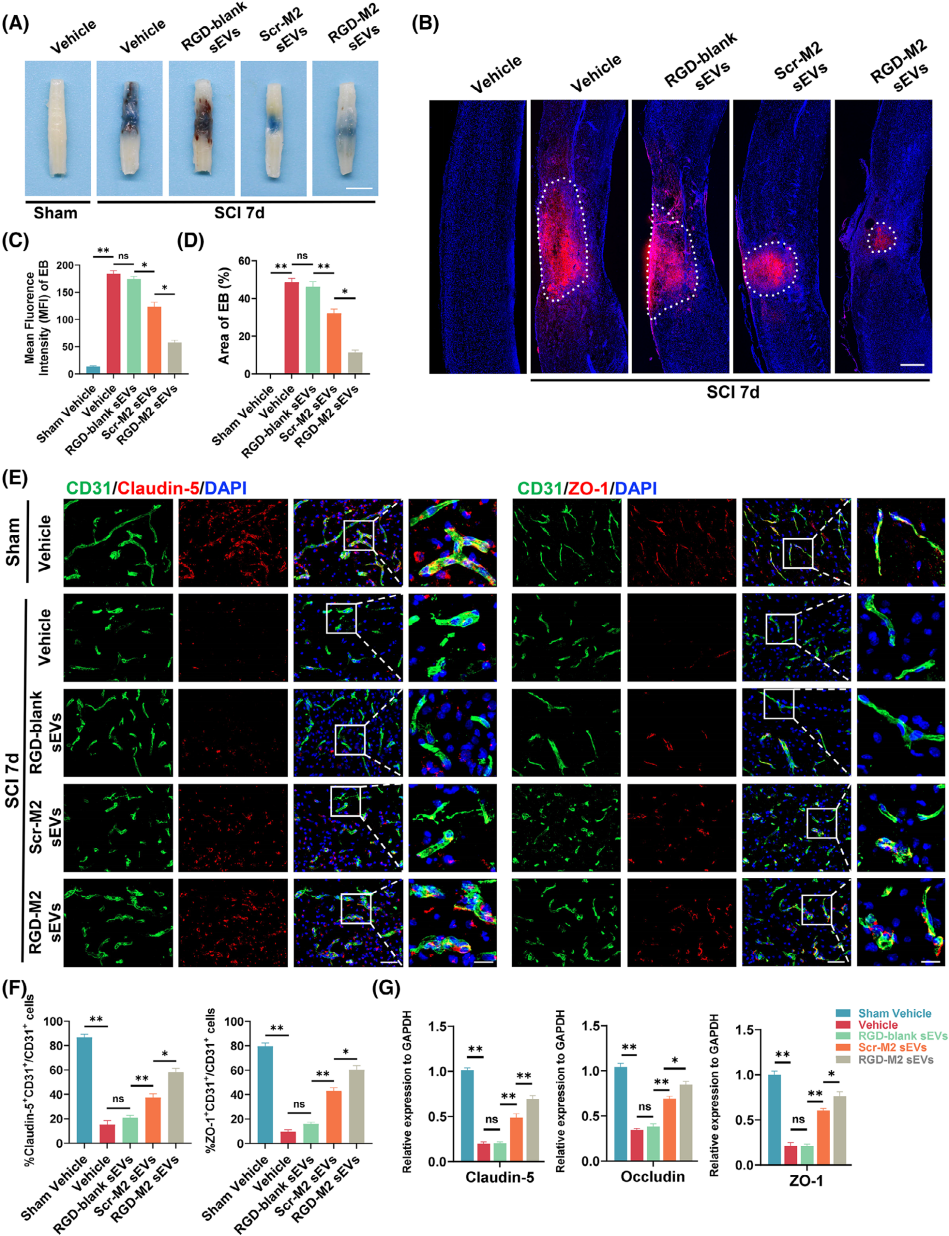
M2 small extracellular vesicles (sEVs) and RGD‐M2 sEVs restore the blood–spinal cord barrier (BSCB) integrity after spinal cord injury (SCI). (A) Digital photographs depicting the appearance of the spinal cords after intravenous injection of Evans blue (EB) into the mice SCI model treated with either Vehicle, RGD‐blank sEVs, Scr‐M2 sEVs or RGD‐M2 sEVs at sham and 7‐day post‐SCI. *n* = 3 per group. Scale bar, 1.0 cm. (B) Immunofluorescence images of the EB extravasation at the injured site in each group on day 7 after SCI. (C) Analysing the EvB fluorescence intensity in (B). *n* = 5 per group. (D) Analysing the area of EvB extravasation in (B). *n* = 5 per group. (E) Immunofluorescence co‐staining of the CD31^+^ endothelial cells (green) and the tight junction‐related protein Claudin‐5 and ZO‐1 (red) at the injured site in each group at 7 days post‐SCI. Scale bar, 50 and 20 µm (enlarged view). (F) Analysing the percentage of CD31^+^Claudin‐5^+^ cells and CD31^+^ZO‐1^+^ cells in CD31^+^ cells in (E). *n* = 5 per group. (G) qPCR verification of the mRNA levels of tight junction‐related protein (Claudin‐5, Occludin and ZO‐1) after the mice treated with Vehicle, RGD‐blank sEVs, Scr‐M2 sEVs or RGD‐M2 sEVs at 7‐day post‐SCI. *n* = 5 per group. ns, no significance, ^*^ *p* < .05, ^**^ *p* < .01. Statistical tests were performed using one‐way analysis of variance (ANOVA) with Tukey's post hoc test (A‒G).

To assess the potential of M2‐sEVs and RGD‐M2‐sEVs in restoring BSCB integrity following SCI, we measured extravasation in the injured region using intravenous administration of EB dye. At 7 days post‐SCI, a peak time for BSCB disruption,^37^ EB leakage was significantly higher in the Vehicle and RGD‐blank sEV groups than in the Scr‐M2‐sEV and RGD‐M2‐sEV groups (Figure 4A). To accurately assess the fluorescence intensity and scope of EB leakage across the groups, fluorescence microscopy was performed. The microscopy results were consistent with digital imaging, demonstrating a dramatic increase in EB extravasation at 7 days post‐SCI in all groups. Nevertheless, the EB extravasation scope was smaller in the RGD‐M2‐sEV group than in the Vehicle, RGD‐blank sEV and Scr‐M2‐sEV groups (Figure 4B–D). Molecular confirmation of these results was obtained through immunofluorescence analysis, which indicated reductions in the post‐SCI protein levels of Claudin‐5 and ZO‐1 in the Vehicle group. However, the fluorescence intensities of these proteins were enhanced in the RGD‐M2‐sEV group compared to those in the Vehicle, Scr‐M2‐sEV and RGD‐blank‐sEV groups (Figure 4E,F). Similarly, qRT‐PCR data showed that the downregulation of TJ mRNA levels in the injured region were remarkably reduced in the RGD‐M2‐sEV group than in the Vehicle, Scr‐M2‐sEV and RGD‐blank‐sEV groups on day 7 post‐SCI (Figure 4G). These preliminary results indicated that both Scr‐M2 and RGD‐M2‐sEVs have the potential to restore BSCB integrity following SCI, with RGD peptide‐decorated M2‐sEVs showing better therapeutic effects.

### 3.5 M2‐sEVs and RGD‐M2‐sEVs elevate perlecan expression in SCMECs and promote post‐SCI locomotive recovery

**FIGURE 5:**
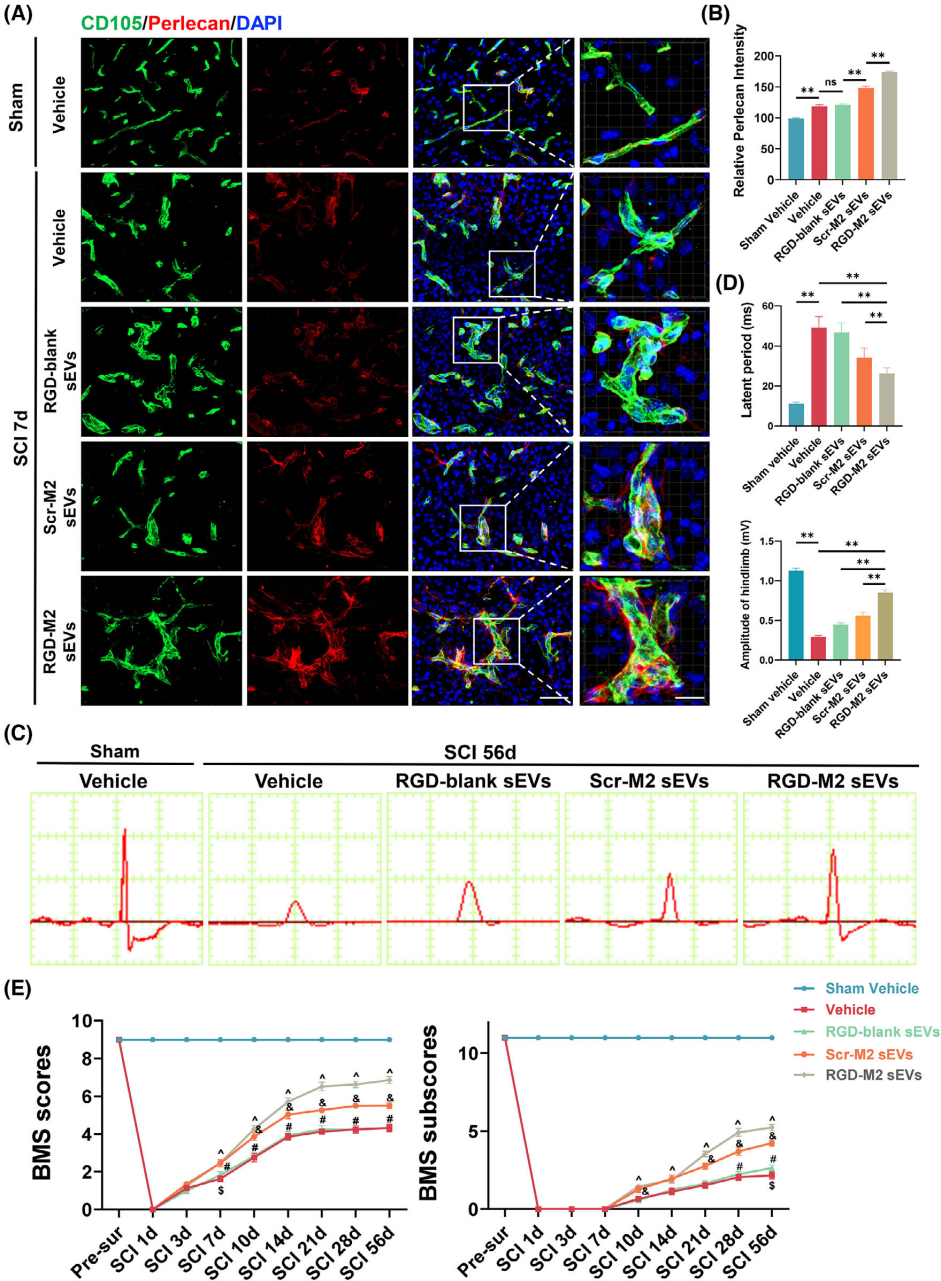
M2 small extracellular vesicles (sEVs) and RGD‐M2 sEVs enhanced the expression of perlecan in spinal cord microvascular endothelial cells (SCMECs) and improved locomotor recovery after spinal cord injury (SCI). (A) Immunofluorescence co‐staining of CD105 (green) and perlecan (red) at the injured site in each group on day 7 post‐SCI. Scale bar, 50 and 20 µm (enlarged view). (B) Analysing the fluorescence intensity of perlecan in (A). *n* = 5 per group. (C) Representative images of motor‐evoked potentials (MEPs) in each group at 56 days post‐SCI. (D) Analysing MEPs' latent period and amplitude in (C). *n* = 6 per group. (E) Distribution of the Basso Mouse Scale (BMS) scores and BMS subscores in each group at pre‐ and post‐SCI throughout the 56 days. *n* = 6 per group. ^^^ *p *< .01 vs. Vehicle group, ^&^ *p* < .05 vs. RGD‐M2 sEVs group, ^#^ *p *< .05 versus RGD‐M2 sEVs group, ^$^ *p* < .05 versus RGD‐blank sEVs group. ns, no significance,^**^ *p* < .01. Statistical tests were performed using one‐way analysis of variance (ANOVA) with Tukey's post hoc test (A‒D) and two‐way ANOVA with Tukey's post hoc test (E).

As a vital component of BMs, perlecan is essential for BSCB maintenance and works in conjunction with other matrix components to support BSCB function overall.^28^, ^38^, ^39^, ^40^ Perlecan overexpression may facilitate reduced BSCB permeability.^28^ Therefore, we performed an immunofluorescence co‐staining analysis to investigate whether the uptake of Scr‐M2‐sEVs and RGD‐M2‐sEVs could increase the level of perlecan in the neovascular endothelium in the injured region. As shown in Figure 5A,B, the perlecan fluorescence intensity in neovascular endothelial cells at the injured area on day 7 post‐SCI was dramatically increased in the RGD‐M2‐sEV group compared with that in the Vehicle, RGD‐blank sEV and Scr‐M2‐sEV groups. This provides evidence that M2‐sEVs, including Scr‐M2‐sEVs and RGD‐M2‐sEVs, increase perlecan expression in neovascular endothelium in the injured area, with RGD‐M2‐sEVs performing better than M2‐sEVs without RGD peptides.

Next, we evaluated whether treatment with Scr‐M2‐sEVs and RGD‐M2‐sEVs impacts the motor function of SCI model mice by performing several behavioural tests, namely, hindlimb electrophysiological analysis and BMS scores and subscores. In hindlimb electrophysiological analysis on day 56 post‐SCI, mice treated with RGD‐M2‐sEVs exhibited markedly greater MEP amplitudes and considerably shorter latent periods than those exposed to Vehicle, RGD‐blank sEVs or Scr‐M2‐sEVs (Figure 5C,D). The results of hindlimb motor function indicated that RGD‐M2‐sEVs significantly enhanced BMS scores and subscores from 7 days up to 56 days post‐SCI. Furthermore, these improvements were much greater than those obtained with Vehicle, RGD‐blank sEVs or Scr‐M2‐sEVs (Figure 5E). On the basis of these behavioural tests, both types of M2‐sEVs—Scr‐M2‐sEVs and RGD‐M2‐sEVs—improved the post‐SCI recovery of neurological functions, with RGD‐M2‐sEVs showing slightly more efficacy than Scr‐M2‐sEVs.

### 3.6 M2‐sEV‐derived perlecan reduces SCMEC permeability in vitro

**FIGURE 6:**
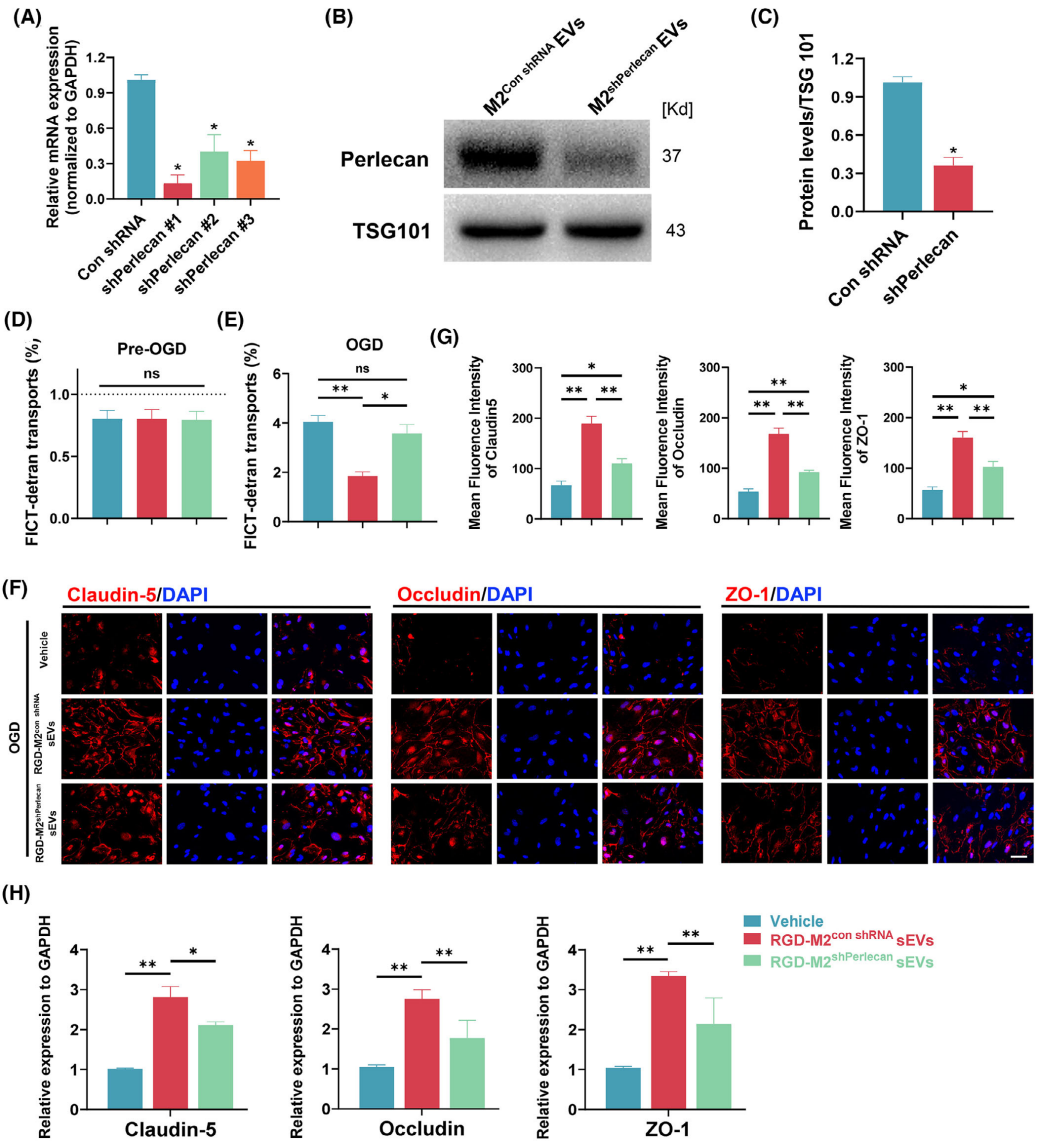
Effect of M2 small extracellular vesicles (sEVs)‐derived perlecan on reducing the permeability of spinal cord microvascular endothelial cells (SCMECs) in vitro. (A) Quantitative real‐time PCR (qRT‐PCR) verified the inhibitory efficiency of three perlecan short hairpin RNAs (shRNAs). (B) Western blotting analysis of perlecan expression in M2^con shRNA^ sEVs and M2^sh^ *^Perlecan^* sEVs groups. *n* = 3 per group. (C) Analysing the perlecan expression levels of (B). *n* = 3 per group. (D and E) FITC‐dextran transport assay to evaluate the permeability of SCMECs treated with Vehicle, RGD‐M2^con shRNA^ sEVs or RGD‐M2^sh^ *^Perlecan^* sEVs before and after oxygen glucose deprivation (OGD). *n* = 5 per group. (F) Immunofluorescence images of the tight junction‐related proteins (Claudin‐5, Occludin and ZO‐1) when SCMECs were treated with Vehicle, RGD‐M2con shRNA sEVs or RGD‐M2sh*Perlecan* sEVs when exposed to OGD. Scale bar, 20 µm. (G) Analysing the fluorescence intensity of Claudin‐5, Occludin and ZO‐1 in (F). *n* = 5 per group. (H) qPCR verification of the mRNA levels of Claudin‐5, Occludin and ZO‐1 in SCMECs treated with Vehicle, RGD‐M2^con shRNA^ sEVs or RGD‐M2^sh^ *^Perlecan^* sEVs post‐OGD. *n* = 5 per group. ns, no significance, ^*^ *p* < .05, ^**^ *p* < .01. Statistical tests were performed using one‐way analysis of variance (ANOVA) with Tukey's post hoc test (A‒H).

To further determine the role of M2‐sEV‐derived perlecan in reducing SCMEC permeability, we designed three shRNAs (sh*Perlecan*#1, sh*Perlecan*#2 and sh*Perlecan*#3) for knockdown of perlecan expression in M2 macrophages. In qRT‐PCR analysis, shPerlecan#1 displayed the most effective inhibition (Figure 6A); thus, it was selected for subsequent experiments. Western blotting confirmed the marked reduction in perlecan levels in M2‐sEVs from M2 macrophages treated with shPerlecan#1 (Figure 6B,C). Next, SCMECs were treated with RGD‐M2‐sEVs from macrophages with perlecan knockdown (RGD‐M2^shPerlecan^ sEVs), control RGD‐M2‐sEVs (RGD‐M2^con shRNA^ sEVs) or Vehicle control. As shown in Figure 6D,E, FITC‐dextran transport analysis revealed a considerable reduction in FITC‐dextran transport in the RGD‐M2^sh^ *^Perlecan^* sEVs group compared to that in the RGD‐M2^con shRNA^ sEVs group. Immunofluorescence analysis of Claudin‐5, Occludin and ZO‐1 in these cells showed repression of these TJ proteins in the RGD‐M2^sh^ *^Perlecan^* sEVs group compared to that in the RGD‐M2^con shRNA^ sEVs group (Figure 6F,G). Similarly to the immunofluorescence results, qRT‐PCR data demonstrated that stimulation of SCMECs with RGD‐M2^sh^ *^Perlecan^* sEVs significantly prevented the increased expression of these TJ proteins compared with RGD‐M2^con shRNA^ sEVs (Figure 6H). These findings indicated that perlecan is essential for the M2‐sEV‐induced reduction in SCMEC permeability in vitro.

### 3.7 M2‐sEV‐derived perlecan promotes BSCB restoration and post‐SCI functional recovery

**FIGURE 7:**
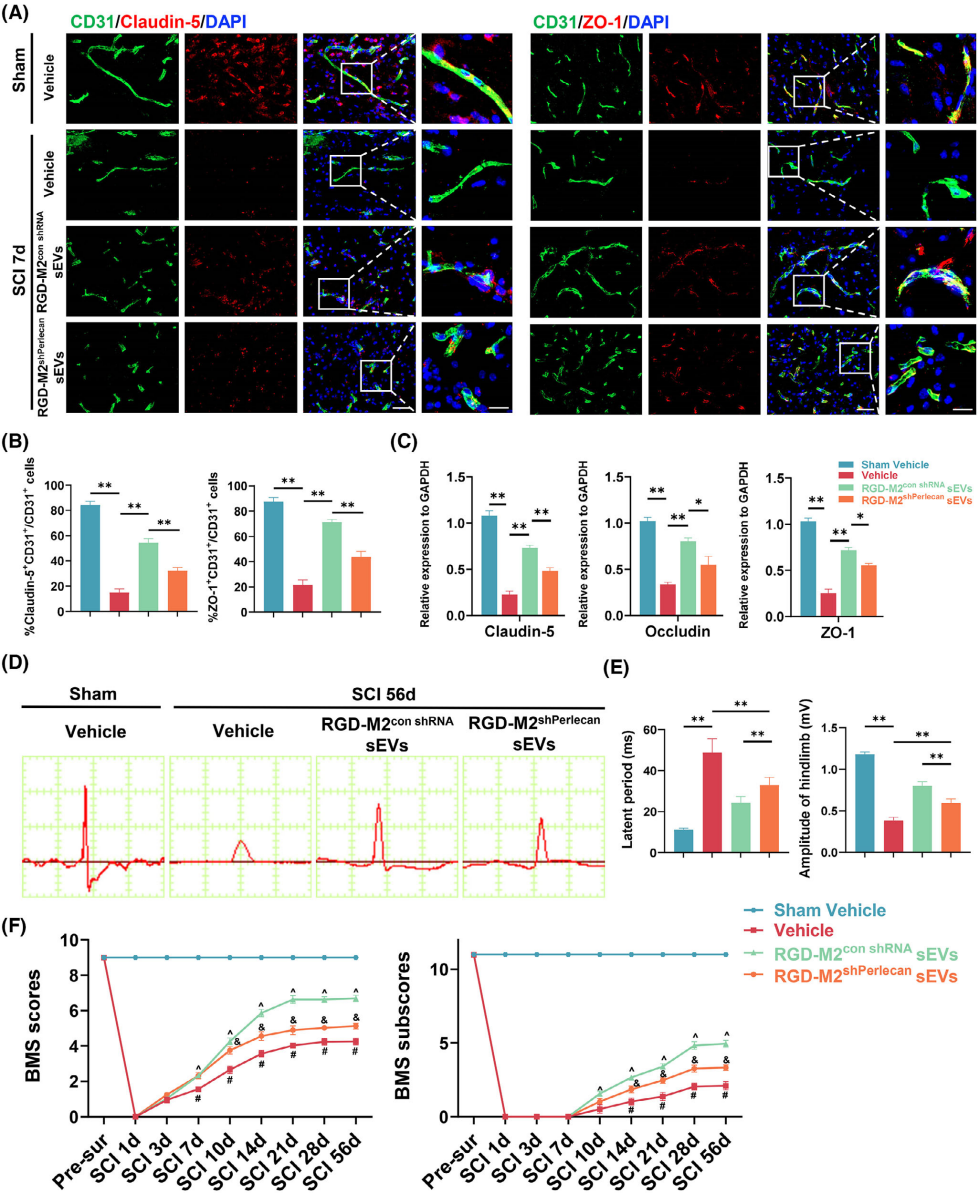
M2 small extracellular vesicle (sEV)‐derived perlecan promotes blood–spinal cord barrier (BSCB) restoration and functional recovery after spinal cord injury (SCI). (A) Immunofluorescence co‐staining of CD31 (green) and tight junction‐related proteins (Claudin‐5 and ZO‐1) (red) at the injured site after treatment with Vehicle, RGD‐M2^con shRNA^ sEVs or RGD‐M2^sh^ *^Perlecan^* sEVs on day 7 post‐SCI. Scale bar, 50 and 20 µm (enlarged view). (B) Analysing the fluorescence intensity of Claudin‐5 and ZO‐1 in (A). *n* = 5 per group. (C) qPCR verification of Claudin‐5, Occludin and ZO‐1 mRNA levels at the injured sits in each group on day 7 after SCI. *n* = 5 per group. (D) Representative images of motor‐evoked potentials (MEPs) in each group at 56 days post‐SCI. (E) Analysing the latent period and amplitude of MEPs in (D). *n* = 6 per group. (F) Distribution of the Basso Mouse Scale (BMS) scores and BMS subscores in each group at pre‐ and post‐SCI throughout the 56 days. *n* = 6 per group. ^^^ *p *< .01 versus Vehicle group, ^&^ *p* < .05 versus RGD‐M2^con shRNA^ sEVs group, ^#^ *p *< .05 versus RGD‐M2^sh^ *^Perlecan^* sEVs group. ns, no significance, ^*^ *p* < .05, ^**^ *p* < .01. Statistical tests were performed using one‐way analysis of variance (ANOVA) with Tukey's post hoc test (A‒E) and two‐way ANOVA with Tukey's post hoc test (F).

Next, we intravenously administered SCI model mice with RGD‐M2^con shRNA^ sEVs, RGD‐M2^shPerlecan^ sEVs, or Vehicle to measure the effectiveness of M2‐sEV‐derived perlecan in promoting BSCB restoration and functional recovery in vivo. Immunofluorescence co‐staining analysis of CD31, Claudin‐5 and ZO‐1 revealed significantly reduced fluorescence intensity in CD31^+^ cells at the injured sites in the RGD‐M2^shPerlecan^ sEVs group compared to that in the RGD‐M2^con shRNA^ sEVs group (Figure 7A,B). Consistently, qRT‐PCR analysis at 7 days post‐SCI demonstrated that, compared with RGD‐M2^con shRNA^ sEVs‐treated mice, the mRNA levels of TJs were suppressed in the injured epicentre of mice treated with RGD‐M2^sh^ *^Perlecan^* sEVs (Figure 7C). In addition, electrophysiological analysis (Figure 7D,E) and BMS scores and subscores (Figure 7F) demonstrated that, compared with the RGD‐M2^con shRNA^ sEVs group, perlecan knockdown in the RGD‐M2^sh^ *^Perlecan^* sEVs group prevented the enhancement of neurological function. These results demonstrated that perlecan was critical for M2‐sEVs‐promoted BSCB restoration and neurological recovery following SCI.

## 4 DISCUSSION

In the present study, we demonstrated that M2‐sEVs were beneficial in restoring the BSCB following SCI in mice. We confirmed our previous proteomics‐based finding of upregulated perlecan expression in M2‐sEVs compared with M2 macrophages, and further showed that M2‐sEVs express high levels of perlecan. Fusion of M2‐sEVs with an RGD peptide at the Lamp2 N‐terminus allowed us to demonstrate the biodistribution of M2‐sEVs and their effects on BSCB restoration in vivo. Furthermore, we discovered that perlecan may contribute significantly to M2‐sEV‐mediated regulation of BSCB integrity and neurological function recovery post‐SCI.

Perlecan, a sizeable multidomain heparan sulphate proteoglycan, was recently shown to be associated with the repair of the BBB and BSCB after CNS injury in several studies.^28^, ^39^, ^40^ Similarly to the BBB, the BSCB primarily comprises SCMECs interconnected by TJs and enveloped within BMs.^4^ It also contains astrocyte end‐feet and pericytes, which extend multiple cytoplasmic processes that surround endothelial cells.^4^ These structures help moderate BSCB fluid dynamics, allowing for the selective passage of specific molecules, such as oxygen and nutrients, providing structural and metabolic support to endothelial cells and neurons, and effectively maintaining local environment homeostasis in the spinal cord.^41^ Perlecan, as a BM component, is a critically significant contributor to the functional recovery from CNS diseases, mediating multiple cellular functions and regulating tissue homeostasis through its interactions with extracellular matrix proteins, growth factors and receptors.^25^, ^26^, ^42^, ^43^ In the present study, we revealed that the culture medium of M2 macrophages reduced SCMEC permeability in an OGD model in vitro. Using an EV inhibition assay, we also found that EVs derived from M2 macrophages played a critical role in restoring SCMEC permeability. We then isolated M2‐sEVs and confirmed that they contained significantly higher levels of perlecan than those in M2 macrophages. In subsequent experiments, we intravenously administered SCI model mice with M2‐sEVs—Scr‐M2‐sEVs or RGD‐M2‐sEVs—and observed that the perlecan levels of the endothelial cells in the injured region were markedly elevated. Both in vitro and in vivo studies showed that M2‐sEVs reduced SCMECs’ permeability and restored BSCB integrity, indicating a therapeutic effect. These findings suggested that perlecan‐enriched M2‐sEVs decrease the permeability of SCMECs.

It is increasingly apparent that EVs have substantial therapeutic potential in the treatment of SCI and traumatic brain injuries. However, their ability to target tissues must first be improved in animal models.^44^, ^45^ Our in vivo data showed that, following SCI, M2‐sEVs did not aggregate in the injured area and showed poor uptake by endothelial cells, indicating limited targeting ability. One approach to conferring sEVs with cell and tissue specificity involves engineering their surface proteins to increase local concentrations of therapeutic factors with minimal side effects.^21^, ^46^, ^47^ Lamp‐2b, which belongs to the lysosome‐associated membrane protein (LAMP) family, is abundantly expressed on the EV surface and slightly expressed on the cell surface, and has been extensively used for targeting EVs by appending targeting sequences.^48^ El‐Andaloussi et al. reported a neuro‐specific exosome that delivers drugs to the CNS and was developed by fusing a rabies viral glycoprotein peptide with the Lamp‐2b protein, which selectively binds to acetylcholine receptors.^49^ In a previous study, we developed neovascular‐specific sEVs targeting the injured spinal cord by decorating M2‐sEVs with RGD peptides fused to the Lamp2b.^17^ RGD peptides bind strongly to integrin α_v_β_3_, which is predominantly expressed in the luminal endothelial cells of neovascular tissues.^36^ Therefore, RGD peptides have received considerable attention for their potential treatment applications in tumours, brain injuries and strokes.^22^, ^23^, ^50^, ^51^ RGD‐decorated EVs are also reportedly effective at targeting and delivering cargo across the BBB to the neovasculature of damaged areas, effectively treating cerebral ischaemic stroke and supporting neurological functional recovery.^22^, ^23^, ^50^ Taledaohan et al. reported on RGD peptide‐decorated nanoparticles incorporating LA‐1, a novel compound designed to target integrin receptor αvβ3. These nanoparticles demonstrated enhanced brain‐targeting efficacy in treating cerebral ischaemia‒reperfusion injury.^51^ Therefore, modifying EVs or nanoparticles with RGD peptides significantly boosts their therapeutic effects, improving traversal of the BBB or BSCB to enhance targeting of damaged areas in the CNS.

Here, we built upon our previous targeting strategy by transducing M2 macrophages with a lentivirus vector encoding an RGD‐tag‐Lamp2 fusion protein, which decorated the surface of the resulting M2‐sEVs. On the basis of TEM and NTA analysis, no statistically significant differences were found in the shape or normal size distribution of M2‐sEVs with and without RGD‐tag‐Lamp2 surface peptides. Furthermore, intravenously administered RGD‐M2‐sEVs showed the ability to target the injured spinal cord area, effectively entering neovascular endothelial cells in SCI model mice. However, the accumulation of RGD‐M2‐sEVs in the liver and spleen was slightly higher than that of Scr‐M2‐sEVs. This may be because sEVs are generally distributed among organs of the mononuclear phagocyte system, with the highest accumulation in the liver, followed by spleen, stomach and lungs. Many intravenously injected compounds, including sEVs, are taken up by patrolling macrophages in the reticuloendothelial system of various organs; for example, Küpffer cells in the liver and macrophages in the spleen.^48^, ^52^ Consequently, in contrast to unmodified sEVs, RGD‐modified sEVs do not enhance the biodistribution of sEVs in other organs.

As in our previous study, we also investigated the uptake of RGD‐sEVs in primary functional cells within the injured area, including glial, macrophages and neuron, following SCI using immunofluorescence co‐staining of Dil‐labelled RGD‐sEVs.^17^ Our findings at 7 days post‐SCI reveal that glials and neurons near the edge of the injured area showed only a slight uptake of Dil‐labelled RGD‐sEVs, whereas macrophages in this area displayed high uptake of Scr‐RGD‐sEVs or RGD‐sEVs. This pronounced uptake may be attributable to the heightened phagocytosis of activated macrophages, which effectively engulf sEVs at the injury site.^53^, ^54^

Extensive research demonstrates the lower immunogenicity of EVs compared with synthetic delivery systems, highlighting a significant advantage in clinical applications.^55^ Furthermore, clinical plasma transfusions are routinely performed, transferring trillions of EVs without adverse events, indicating the biocompatibility of allogeneic EVs.^52^, ^56^ Animal studies have revealed that the administration of human‐derived EVs does not lead to immune reactions or toxicity.^57^ Major histocompatibility complex molecules, which are enriched on EVs, are the main source of antigens that trigger xenogeneic and allogeneic immune responses.^58^, ^59^ Compared with chemical engineering and membrane hybridisation techniques for modifying EVs, genetic engineering approaches do not increase the immunogenicity of EVs.^60^, ^61^ This may be because such genetically engineered proteins are fused with an EV membrane protein without altering the overall properties of the EV membrane.^62^, ^63^ The current study found no significant immunological side effects for either the Scr‐M2‐sEVs or RGD‐M2‐sEVs in mice.

In recent years, advancements in targeted peptides and materials have opened up exciting new possibilities, including the innovative CP05 anchor peptide and magnetic targeting technologies.^64^ However, CP05 does not have targeting capability, requiring linkage with other targeted peptides to achieve specific targeting.^65^ This requirement increases the complexity of creating delivery platforms and the number of transductions, thereby reducing transduction efficiency. Magnetic targeting materials for drug delivery are often used with microspheres or nanoparticles, with superparamagnetic iron oxide nanoparticles (SPION) being the most common example. A magnet is positioned near the surface of the target organ to guide the magnetic drug delivery system toward that specific area. However, the ability of these materials to target a particular type of tissue or cell population may not be precise enough. By contrast, RGD‐decorated M2‐sEVs show minimal binding to other cell types, thus reducing potential harmful effects on surrounding cells.

Engineering modifications to confer EVs with exceptional features, such as targeting ability and overexpression of specific genes, is currently a research hotspot. Apart from our use of RGD modified EVs for the precise treatment of the BSCB, Kong et al. reported modifying M2 microglia‐derived EVs with the angiopep‐2 oligopeptide, which successfully targeted injured cells of the spinal cord.^66^ These EVs were primarily taken up by microglia and other macrophages; however, these are not the main cell types in the BSCB. Here, we have used RGD peptides to effectively target neovascular endothelial cells, which are the primary cells in BSCB repair post‐SCI, playing a crucial role in regulating permeability.

We also investigated the impacts of both RGD‐M2‐sEVs and Scr‐M2‐sEVs on BSCB repair in SCI model mice. While both types of M2‐sEVs enhanced the expression of critical BSCB‐associated proteins, thus restoring BSCB integrity and improving neurological function in vitro and in vivo, RGD‐M2‐sEVs provided superior efficacy. These results highlighted the potential of RGD‐M2‐sEVs as a neovascular agent in BSCB repair and functional recovery from SCI.

However, EVs contain various components, including proteins, bioactive lipids and nucleic acids, which can trigger biological responses in recipient cells.^14^ To confirm whether the perlecan‐mediated reduction in SCMEC permeability is necessary for restoring the BSCB, we tested RGD‐M2‐sEVs with perlecan knockdown. This knockdown significantly inhibited the trend towards reduced vascular and BSCB permeability and increased functional recovery in vitro and in vivo. Nevertheless, it is worth noting that RGD‐M2‐sEVs with perlecan knockdown retained the ability to promote functional recovery following SCI. Additionally, our previous research suggested that the OTULIN protein in M2‐sEVs contributed to vascular regeneration, possibly explaining their influence on neurological recovery.^24^ Therefore, multiple molecules in M2‐sEVs may be associated with BSCB repair and neural regeneration, necessitating further research to clarify their functions.

In our study, we found that RGD‐M2‐sEVs delivered perlecan to the neovascular endothelium in the injured area, likely speeding up SCI recovery. Recently, the specific signalling pathway through which perlecan helps restore the BSCB was identified. Kuniyuki et al. discovered that Hspg2‐deficient mice (*Hspg2* ^−/−^‐TG) with conditional perlecan expression had larger infarct volumes and more BBB leakage than control mice in a transient middle cerebral artery occlusion model.^39^ Their findings indicated that perlecan is crucial in regulating pericyte recruitment through the coordinated functioning of PGFR‐β and integrin α_5_β_1_, supporting BBB maintenance and repair after ischaemic stroke.^39^ Furthermore, Xie et al. found that overexpressing perlecan decreases BSCB permeability and neuroinflammatory responses, thereby enhancing locomotor recovery and neuronal regeneration in mice with SCI.^28^ They demonstrated that, in addition to inhibiting the formation of stress fibres through interaction with integrin β1, perlecan also inhibits the downstream ROCK/MLC signalling. This action decreases the disassembly of TJs (e.g., ZO‐1 and Occludin) and enhances the integrity of the BBB (BSCB).^28^ These studies offer an in‐depth understanding of the molecular mechanisms by which perlecan contributes to BSCB repair, ultimately enhancing post‐SCI neurological function. This insight underscores the critical role of perlecan in recovery, highlighting its potential as a key therapeutic target.

Overall, our results supported the observation that perlecan is abundant in M2 macrophages and enriched in M2‐sEVs. Furthermore, we provided corroborated evidence that M2‐sEVs can reduce BSCB permeability and reestablish BSCB integrity, highlighting their promise as a treatment agent for SCI. To enhance the local drug concentration and targetability of perlecan, the surface of M2‐sEVs was modified with RGD peptides fused to N‐terminal Lamp2. These engineered RGD‐M2‐sEVs accumulated at the site of injury, positively influencing BSCB restoration and improving neurological function in SCI model mice. RGD‐M2‐sEVs may represent a potential treatment strategy for SCI.

This study has some limitations. First, the mechanisms and severity of SCI in humans often differ from those in animal models, making it challenging to establish a model that fully replicates all aspects of acute human SCI. Consequently, some results from this study may not accurately reflect real‐world conditions in patients. Second, we discovered that recovery of post‐SCI neurological function is generally better in small vertebrate animals than in humans. This difference may have influenced the experimental outcomes and introduced bias. Third, although the number of experimental animals used in our study met the required standards, the smaller sample size compared with clinical studies and potential statistical bias limited our ability to fully assess possible imbalances in the results. Finally, our experiments were not validated in larger primates, such as rhesus monkeys, which is essential for preclinical research, highlighting a limitation that future studies may need to address.

## 5 CONCLUSION

Our study demonstrates that targeted delivery of perlecan‐enriched sEVs derived from M2 macrophages, modified with RGD peptides for enhanced accumulation, improves therapeutic efficacy in SCI. This approach advances the understanding of BSCB repair and offers a novel therapeutic strategy for SCI treatment.

## AUTHOR CONTRIBUTIONS

*Conceptualisation, writing—original draft and supervision*: Yong Xie. *Funding acquisition, project administration and formal analysis*: Wei Peng. *Funding acquisition, data curation, formal analysis and writing—original draft*: Shujun Zhang. *Data curation and formal analysis*: Wentao Zhang. *Data curation and formal analysis*: Wei Cui. *Investigation, methodology and validation*: Wenjin Chen. *Investigation, resources and methodology*: Yin Zhuang. *Writing and editing, and visualisation*: Rupeng Chu. *Data curation*: Jinghua Tan. *Writing—review and editing*: Jingbo Xue. *Supervision*: Yiguo Yan. *Investigation*: Guoyong Yin.

## CONFLICT OF INTEREST STATEMENT

The authors declare that there is no conflict of interest regarding the publication of this paper.

## ETHICS STATEMENT

The Ethics Committee of Wuxi ninth Affiliated Hospital of Soochow University for Scientific Research has approved all experimental animal procedures (KS2024057).

## Supporting information



Supporting Information

Supporting Information

Supporting Information

## Data Availability

The data that support the findings of this study are available from the corresponding author upon reasonable request.

## References

[1] Eckert MJ , Martin MJ . Trauma: spinal cord injury. Surg Clin North Am. 2017;97(5):1031‐1045. 28958356 10.1016/j.suc.2017.06.008

[2] Moshi H , Sundelin G , Sahlen K‐G , Sörlin A . Traumatic spinal cord injury in the north‐east Tanzania—describing incidence, etiology and clinical outcomes retrospectively. Glob Health Action. 2017;10(1):1355604. 28856978 10.1080/16549716.2017.1355604 PMC5645664

[3] Yu Q , Huang J , Hu J , Zhu H . Advance in spinal cord ischemia reperfusion injury: blood‒spinal cord barrier and remote ischemic preconditioning. Life Sci. 2016;154:34‐38. 27060223 10.1016/j.lfs.2016.03.046

[4] Bartanusz V , Jezova D , Alajajian B , Digicaylioglu M . The blood‒spinal cord barrier: morphology and clinical implications. Ann Neurol. 2011;70(2):194‐206. 21674586 10.1002/ana.22421

[5] Altinova H , Hammes S , Palm M , et al. Dense fibroadhesive scarring and poor blood vessel‐maturation hamper the integration of implanted collagen scaffolds in an experimental model of spinal cord injury. Biomed Mater. 2020;15(1):015012. 31796648 10.1088/1748-605X/ab5e52

[6] Jin LY, Li J , Wang KF , et al. Blood‒spinal cord barrier in spinal cord injury: a review. J Neurotrauma. 2021;38(9):1203‐1224. 33292072 10.1089/neu.2020.7413

[7] Figley SA , Khosravi R , Legasto JM , Tseng YF , Fehlings MG . Characterization of vascular disruption and blood‐spinal cord barrier permeability following traumatic spinal cord injury. J Neurotrauma. 2014;31(6):541‐552. 24237182 10.1089/neu.2013.3034 PMC3949504

[8] Takigawa T , Yonezawa T , Yoshitaka T , et al. Separation of the perivascular basement membrane provides a conduit for inflammatory cells in a mouse spinal cord injury model. J Neurotrauma. 2010;27(4):739‐751. 20038195 10.1089/neu.2009.1111

[9] Tator CH , Fehlings MG . Review of the secondary injury theory of acute spinal cord trauma with emphasis on vascular mechanisms. J Neurosurg. 1991;75(1):15‐26. 2045903 10.3171/jns.1991.75.1.0015

[10] Hemley SJ , Tu J , Stoodley MA . Role of the blood‒spinal cord barrier in posttraumatic syringomyelia. J Neurosurg Spine. 2009;11(6):696‐704. 19951022 10.3171/2009.6.SPINE08564

[11] Jiang T , Qin T , Gao P , et al. SIRT1 attenuates blood‒spinal cord barrier disruption after spinal cord injury by deacetylating p66Shc. Redox Biol. 2023;60:102615. 36716673 10.1016/j.redox.2023.102615 PMC9900454

[12] Willenborg S , Lucas T , van Loo G , et al. CCR2 recruits an inflammatory macrophage subpopulation critical for angiogenesis in tissue repair. Blood. 2012;120(3):613‐625. 22577176 10.1182/blood-2012-01-403386

[13] Zajac E , Schweighofer B , Kupriyanova TA , et al. Angiogenic capacity of M1‐ and M2‐polarized macrophages is determined by the levels of TIMP‐1 complexed with their secreted proMMP‐9. Blood. 2013;122(25):4054‐4067. 24174628 10.1182/blood-2013-05-501494 PMC3862278

[14] Fruhbeis C , Frohlich D , Kuo WP , et al. Neurotransmitter‐triggered transfer of exosomes mediates oligodendrocyte‐neuron communication. PLoS Biol. 2013;11(7):e1001604. 23874151 10.1371/journal.pbio.1001604 PMC3706306

[15] Kalluri R , LeBleu VS . The biology, function, and biomedical applications of exosomes. Science. 2020;367(6478):eaau6977. 32029601 10.1126/science.aau6977 PMC7717626

[16] Liu W , Wang Y , Gong F , et al. Exosomes derived from bone mesenchymal stem cells repair traumatic spinal cord injury by suppressing the activation of A1 neurotoxic reactive astrocytes. J Neurotrauma. 2019;36(3):469‐484. 29848167 10.1089/neu.2018.5835

[17] Peng W , Xie Y , Liu Y , et al. Targeted delivery of CD163(+) macrophage‐derived small extracellular vesicles via RGD peptides promote vascular regeneration and stabilization after spinal cord injury. J Control Release. 2023;361:750‐765. 37586563 10.1016/j.jconrel.2023.08.025

[18] Peng W , Xie Y , Luo Z , et al. UTX deletion promotes M2 macrophage polarization by epigenetically regulating endothelial cell‐macrophage crosstalk after spinal cord injury. J Nanobiotechnol. 2023;21(1):225. 10.1186/s12951-023-01986-0 PMC10350278 37454119

[19] Wang J , Rong Y , Ji C , et al. MicroRNA‐421‐3p‐abundant small extracellular vesicles derived from M2 bone marrow‐derived macrophages attenuate apoptosis and promote motor function recovery via inhibition of mTOR in spinal cord injury. J Nanobiotechnol. 2020;18(1):72. 10.1186/s12951-020-00630-5 PMC7222346 32404105

[20] Zeng J , Gu C , Sun Y , Chen X . Engineering of M2 macrophages‐derived exosomes via click chemistry for spinal cord injury repair. Adv Healthc Mater. 2023;12(11):e2203391. 36877863 10.1002/adhm.202203391

[21] Tian T , Zhang HX , He CP , et al. Surface functionalized exosomes as targeted drug delivery vehicles for cerebral ischemia therapy. Biomaterials. 2018;150:137‐149. 29040874 10.1016/j.biomaterials.2017.10.012

[22] Tian T , Cao L , He C , et al. Targeted delivery of neural progenitor cell‐derived extracellular vesicles for anti‐inflammation after cerebral ischemia. Theranostics. 2021;11(13):6507‐6521. 33995671 10.7150/thno.56367 PMC8120222

[23] Zhang H , Wu J , Wu J , et al. Exosome‐mediated targeted delivery of miR‐210 for angiogenic therapy after cerebral ischemia in mice. J Nanobiotechnol. 2019;17(1):29. 10.1186/s12951-019-0461-7 PMC6379944 30782171

[24] Luo Z , Peng W , Xu Y , et al. Exosomal OTULIN from M2 macrophages promotes the recovery of spinal cord injuries via stimulating Wnt/β‐catenin pathway‐mediated vascular regeneration. Acta Biomater. 2021;136:519‐532. 34551329 10.1016/j.actbio.2021.09.026

[25] Thomsen MS , Routhe LJ , Moos T . The vascular basement membrane in the healthy and pathological brain. J Cereb Blood Flow Metab. 2017;37(10):3300‐3317. 28753105 10.1177/0271678X17722436 PMC5624399

[26] Roberts J , Kahle MP , Bix GJ . Perlecan and the blood‒brain barrier: beneficial proteolysis? Front Pharmacol. 2012;3:155. 22936915 10.3389/fphar.2012.00155 PMC3425914

[27] Lee B , Clarke D , Al Ahmad A , et al. Perlecan domain V is neuroprotective and proangiogenic following ischemic stroke in rodents. J Clin Invest. 2011;121(8):3005‐3023. 21747167 10.1172/JCI46358 PMC3148740

[28] Xie C , Wang Y , Wang J , et al. Perlecan improves blood spinal cord barrier repair through the integrin β1/ROCK/MLC pathway after spinal cord injury. Mol Neurobiol. 2023;60(1):51‐67. 36216996 10.1007/s12035-022-03041-9

[29] Ruck T , Bittner S , Epping L , Herrmann AM , Meuth SG . Isolation of primary murine brain microvascular endothelial cells. J Vis Exp. 2014;(93):e52204. 25489873 10.3791/52204 PMC4354020

[30] Théry C , Amigorena S , Raposo G , Clayton A . Isolation and characterization of exosomes from cell culture supernatants and biological fluids. Curr Protoc Cell Biol. 2006. Chapter 3:Unit 3.22. 10.1002/0471143030.cb0322s30 18228490

[31] Liu J , Jin X , Liu KJ , Liu W . Matrix metalloproteinase‐2‐mediated occludin degradation and caveolin‐1‐mediated claudin‐5 redistribution contribute to blood–brain barrier damage in early ischemic stroke stage. J Neurosci. 2012;32(9):3044‐3057. 22378877 10.1523/JNEUROSCI.6409-11.2012 PMC3339570

[32] Ge X , Tang P , Rong Y , et al. Exosomal miR‐155 from M1‐polarized macrophages promotes EndoMT and impairs mitochondrial function via activating NF‐kappaB signaling pathway in vascular endothelial cells after traumatic spinal cord injury. Redox Biol. 2021;41:101932. 33714739 10.1016/j.redox.2021.101932 PMC7967037

[33] Basso DM , Fisher LC , Anderson AJ , Jakeman LB , McTigue DM , Popovich PG . Basso Mouse Scale for locomotion detects differences in recovery after spinal cord injury in five common mouse strains. J Neurotrauma. 2006;23(5):635‐659. 16689667 10.1089/neu.2006.23.635

[34] Schlag MG , Hopf R , Redl H . Serial recording of sensory, corticomotor, and brainstem‐derived motor evoked potentials in the rat. Somatosens Motor Res. 2001;18(2):106‐116. 10.1080/135578501012006219 11534774

[35] Tkach M , Thery C . Communication by extracellular vesicles: where we are and where we need to go. Cell. 2016;164(6):1226‐1232. 26967288 10.1016/j.cell.2016.01.043

[36] Ley K , Rivera‐Nieves J , Sandborn WJ , Shattil S . Integrin‐based therapeutics: biological basis, clinical use and new drugs. Nat Rev Drug Discov. 2016;15(3):173‐183. 26822833 10.1038/nrd.2015.10 PMC4890615

[37] Whetstone WD , Hsu J‐YC , Eisenberg M , Werb Z , Noble‐Haeusslein LJ . Blood–spinal cord barrier after spinal cord injury: relation to revascularization and wound healing. J Neurosci Res. 2003;74(2):227‐239. 14515352 10.1002/jnr.10759 PMC2837839

[38] Arikawa‐Hirasawa E , Watanabe H , Takami H , Hassell JR , Yamada Y . Perlecan is essential for cartilage and cephalic development. Nat Genet. 1999;23(3):354‐358. 10545953 10.1038/15537

[39] Nakamura K , Ikeuchi T , Nara K , et al. Perlecan regulates pericyte dynamics in the maintenance and repair of the blood‐brain barrier. J Cell Biol. 2019;218(10):3506‐3525. 31541017 10.1083/jcb.201807178 PMC6781430

[40] Xu L , Nirwane A , Yao Y . Basement membrane and blood‐brain barrier. Stroke Vasc Neurol. 2019;4(2):78‐82. 31338215 10.1136/svn-2018-000198 PMC6613871

[41] Hu J , Yu Q , Xie L , Zhu H . Targeting the blood–spinal cord barrier: a therapeutic approach to spinal cord protection against ischemia–reperfusion injury. Life Sci. 2016;158:1‐6. 27329433 10.1016/j.lfs.2016.06.018

[42] Kerever A , Mercier F , Nonaka R , et al. Perlecan is required for FGF‐2 signaling in the neural stem cell niche. Stem Cell Res. 2014;12(2):492‐505. 24434631 10.1016/j.scr.2013.12.009 PMC3952240

[43] Martinez JR , Dhawan A , Farach‐Carson MC . Modular proteoglycan Perlecan/HSPG2: mutations, phenotypes, and functions. Genes (Basel). 2018;9(11):556. 10.3390/genes9110556 PMC6266596 30453502

[44] Xu M , Feng T , Liu B , et al. Engineered exosomes: desirable target‐tracking characteristics for cerebrovascular and neurodegenerative disease therapies. Theranostics. 2021;11(18):8926‐8944. 34522219 10.7150/thno.62330 PMC8419041

[45] Nakazaki M , Morita T , Lankford KL , Askenase PW , Kocsis JD . Small extracellular vesicles released by infused mesenchymal stromal cells target M2 macrophages and promote TGF‐β upregulation, microvascular stabilization and functional recovery in a rodent model of severe spinal cord injury. J Extracell Vesicles. 2021;10(11):e12137. 34478241 10.1002/jev2.12137 PMC8408371

[46] Bai J , Duan J , Liu R , et al. Engineered targeting tLyp‐1 exosomes as gene therapy vectors for efficient delivery of siRNA into lung cancer cells. Asian J Pharm Sci. 2020;15(4):461‐471. 32952669 10.1016/j.ajps.2019.04.002 PMC7486479

[47] Cao Y , Wu T , Zhang K , et al. Engineered exosome‐mediated near‐infrared‐II region V(2)C quantum dot delivery for nucleus‐target low‐temperature photothermal therapy. ACS Nano. 2019;13(2):1499‐1510. 30677286 10.1021/acsnano.8b07224

[48] Liang Y , Duan L , Lu J , Xia J . Engineering exosomes for targeted drug delivery. Theranostics. 2021;11(7):3183‐3195. 33537081 10.7150/thno.52570 PMC7847680

[49] El‐Andaloussi S , Lee Y , Lakhal‐Littleton S , et al. Exosome‐mediated delivery of siRNA in vitro and in vivo. Nat Protoc. 2012;7(12):2112‐2126. 23154783 10.1038/nprot.2012.131

[50] Liang HB , Chen X , Zhao R , et al. Simultaneous ischemic regions targeting and BBB crossing strategy to harness extracellular vesicles for therapeutic delivery in ischemic stroke. J Control Release. 2024;365:1037‐1057. 38109946 10.1016/j.jconrel.2023.12.021

[51] Taledaohan A , Tuohan MM , Jia R , et al. An RGD‐conjugated prodrug nanoparticle with blood–brain‐barrier penetrability for neuroprotection against cerebral ischemia–reperfusion injury. Antioxidants (Basel). 2024;13(11):1339. 10.3390/antiox13111339 PMC11591307 39594481

[52] Kimiz‐Gebologlu I , Oncel SS . Exosomes: large‐scale production, isolation, drug loading efficiency, and biodistribution and uptake. J Control Release. 2022;347:533‐543. 35597405 10.1016/j.jconrel.2022.05.027

[53] Wiklander OPB , Nordin JZ , O'Loughlin A , et al. Extracellular vesicle in vivo biodistribution is determined by cell source, route of administration and targeting. J Extracell Vesicles. 2015;4:26316. 25899407 10.3402/jev.v4.26316 PMC4405624

[54] Charoenviriyakul C , Takahashi Y , Morishita M , Matsumoto A , Nishikawa M , Takakura Y . Cell type‐specific and common characteristics of exosomes derived from mouse cell lines: yield, physicochemical properties, and pharmacokinetics. Eur J Pharmaceut Sci. 2017;96:316‐322. 10.1016/j.ejps.2016.10.009 27720897

[55] Xia Y , Zhang J , Liu G , Wolfram J . Immunogenicity of extracellular vesicles. Adv Mater. 2024;36(33):e2403199. 38932653 10.1002/adma.202403199

[56] Witwer KW , Wolfram J . Extracellular vesicles versus synthetic nanoparticles for drug delivery. Nat Rev Mater. 2021;6(2):103‐106. 36117545 10.1038/s41578-020-00277-6 PMC9481198

[57] Zhu X , Badawi M , Pomeroy S , et al. Comprehensive toxicity and immunogenicity studies reveal minimal effects in mice following sustained dosing of extracellular vesicles derived from HEK293T cells. J Extracell Vesicles. 2017;6(1):1324730. 28717420 10.1080/20013078.2017.1324730 PMC5505007

[58] Zeng F , Chen Z , Chen R , et al. Graft‐derived extracellular vesicles transported across subcapsular sinus macrophages elicit B cell alloimmunity after transplantation. Sci Transl Med. 2021;13(585):eabb0122. 33731430 10.1126/scitranslmed.abb0122 PMC8939235

[59] Bauzá‐Martinez J , Heck AJR , Wu W . HLA‐B and cysteinylated ligands distinguish the antigen presentation landscape of extracellular vesicles. Commun Biol. 2021;4(1):825. 34211107 10.1038/s42003-021-02364-y PMC8249458

[60] Yang C , Xue Y , Duan Y , Mao C , Wan M . Extracellular vesicles and their engineering strategies, delivery systems, and biomedical applications. J Control Release. 2024;365:1089‐1123. 38065416 10.1016/j.jconrel.2023.11.057

[61] Sun M , Yang J , Fan Y , et al. Beyond extracellular vesicles: hybrid membrane nanovesicles as emerging advanced tools for biomedical applications. Adv Sci (Weinh). 2023;10(32):e2303617. 37749882 10.1002/advs.202303617 PMC10646251

[62] Xu F , Fei Z , Dai H , et al. Mesenchymal stem cell‐derived extracellular vesicles with high PD‐L1 expression for autoimmune diseases treatment. Adv Mater. 2022;34(1):e2106265. 34613627 10.1002/adma.202106265

[63] Yang Y , Hong Y , Cho E , Kim GB , Kim IS . Extracellular vesicles as a platform for membrane‐associated therapeutic protein delivery. J Extracell Vesicles. 2018;7(1):1440131. 29535849 10.1080/20013078.2018.1440131 PMC5844050

[64] Ahn W , Han J , Kim N , et al. Hierarchical protein nano‐crystalline hydrogel with extracellular vesicles for ectopic lymphoid structure formation. Biomaterials. 2025;318:123166. 39933315 10.1016/j.biomaterials.2025.123166

[65] Gao X , Ran N , Dong X , et al. Anchor peptide captures, targets, and loads exosomes of diverse origins for diagnostics and therapy. Sci Transl Med. 2018;10(444):eaat0195. 10.1126/scitranslmed.aat0195 29875202

[66] Kong G , Liu J , Wang J , et al. Engineered extracellular vesicles modified by angiopep‐2 peptide promote targeted repair of spinal cord injury and brain inflammation. ACS Nano. 2025;19(4):4582‐4600. 39853366 10.1021/acsnano.4c14675 PMC11803916

