# Supplementary figures and images for: Engineered small extracellular vesicles for targeted delivery of perlecan to stabilise the blood–spinal cord barrier after spinal cord injury
Source: Clin Transl Med. 2025 Jun 19;15(6):e70381. doi: 10.1002/ctm2.70381 (PMC12179339; doi:10.1002/ctm2.70381)

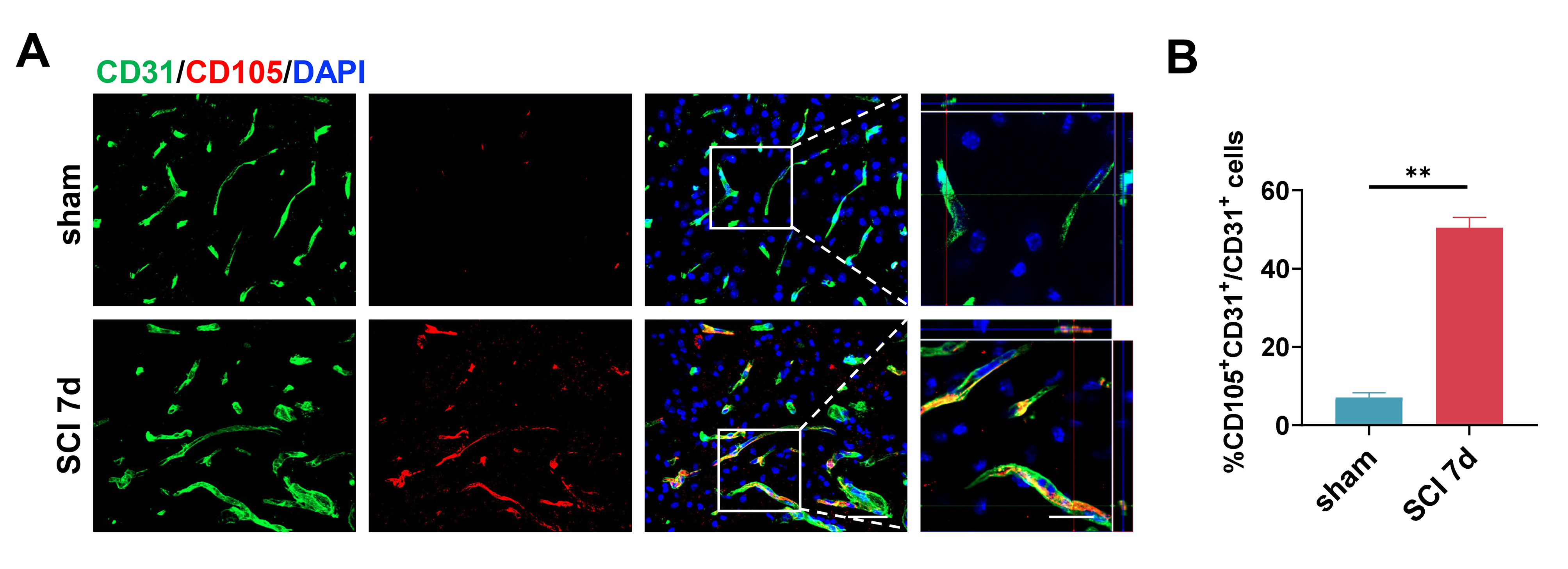

Supplement: Supplementary file 1 — Supporting Information [file CTM2-15-e70381-s001.png]

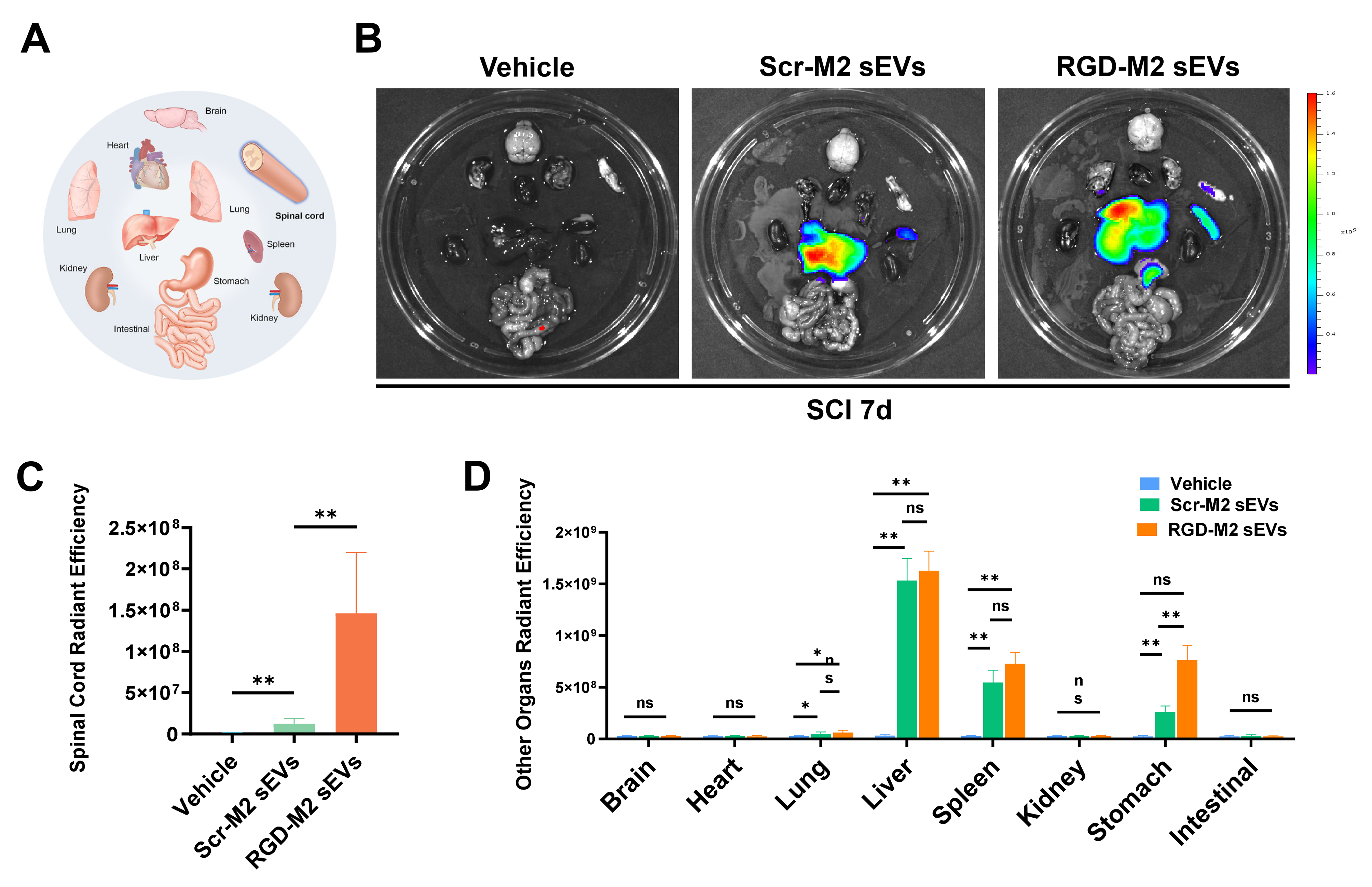

Supplement: Supplementary file 2 — Supporting Information [file CTM2-15-e70381-s002.png]

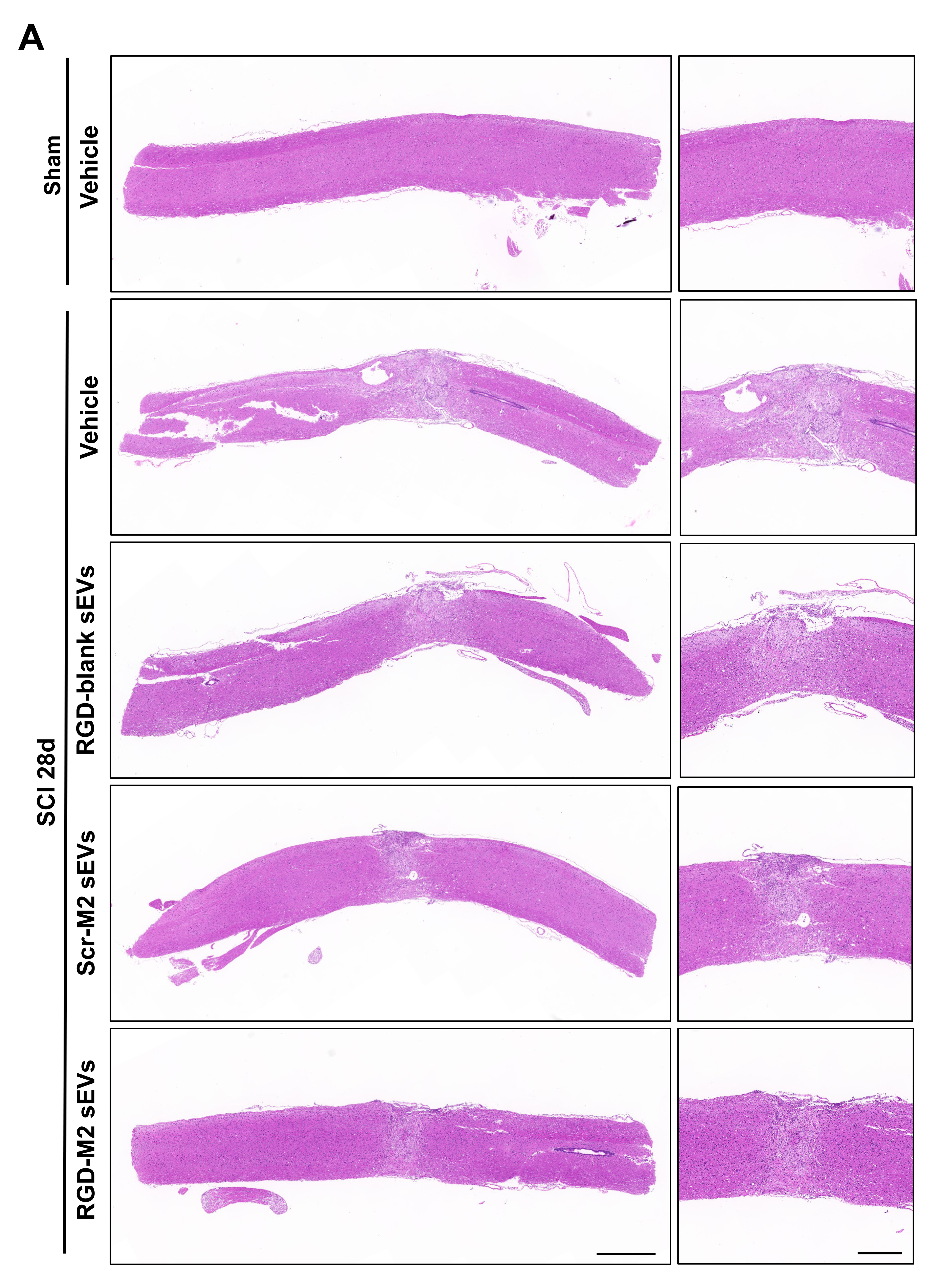

Supplement: Supplementary file 3 — Supporting Information [file CTM2-15-e70381-s003.png]
